# Li_2_CO_3_-affiliative mechanism for air-accessible interface engineering of garnet electrolyte via facile liquid metal painting

**DOI:** 10.1038/s41467-020-17493-x

**Published:** 2020-07-24

**Authors:** Junwei Meng, Yang Zhang, Xuejun Zhou, Meng Lei, Chilin Li

**Affiliations:** 1 State Key Laboratory of High Performance Ceramics and Superfine Microstructure, Shanghai Institute of Ceramics, Chinese Academy of Sciences, 585 He Shuo Road, 201899 Shanghai, China; 2 Center of Materials Science and Optoelectronics Engineering, University of Chinese Academy of Sciences, 100049 Beijing, China

**Keywords:** Batteries, Batteries, Batteries

## Abstract

Garnet based solid-state batteries have the advantages of wide electrochemical window and good chemical stability. However, at Li-garnet interface, the poor interfacial wettability due to Li_2_CO_3_ passivation usually causes large resistance and unstable contact. Here, a Li_2_CO_3_-affiliative mechanism is proposed for air-accessible interface engineering of garnet electrolyte via facile liquid metal (LM) painting. The natural LM oxide skin enables a superior wettability of LM interlayer towards ceramic electrolyte and Li anode. Therein the removal of Li_2_CO_3_ passivation network is not necessary, in view of its delamination and fragmentation by LM penetration. This dissipation effect allows the lithiated LM nanodomains to serve as alternative Li-ion flux carriers at Li-garnet interface. This mechanism leads to an interfacial resistance as small as 5 Ω cm^2^ even after exposing garnet in air for several days. The ultrastable Li plating and stripping across LM painted garnet can last for 9930 h with a small overpotential.

## Abstract (web summary)

At Li-garnet interface, the poor interfacial wettability due to Li_2_CO_3_ passivation causes large resistance and unstable contact. Here the authors propose a Li_2_CO_3_-affiliative mechanism for air-accessible interface engineering of garnet electrolyte with superior wettability via facile liquid metal painting.

## Introduction

Lithium metal batteries (LMBs) are attracting more attentions due to their high energy densities, benefitting from lithium metal anode with low redox electrochemical potential and high theoretical specific capacity^1,2^. Uncontrollable lithium dendrite growth would cause serious side reaction with organic liquid electrolyte (LE) and even lead to the dry up of electrolyte. The inferior cycling performance as well as the high safety risk (such as leakage and explosion) severely hinder the development and wide application of LMBs^3,4^. The employment of solid-state electrolyte (SSE) is thought to be a promising solution to Li dendrite suppression due to its high Young’s modulus (e.g., ~150 GPa for garnet oxide ceramic) and chemical stability even at high temperature (e.g., up to 300 °C for garnet)^5,6^. Besides, many SSEs possess broader electrochemical window which can reach up to 5 V^6,7^. Among current SSEs, polymer or hybrid electrolytes provide the advantages of flexibility and deformability but at a cost of relatively low ionic conductivity at room temperature (RT) (<0.1 mS cm^−1^)^8,9^. Inorganic ceramic SSEs not only provide high ionic conductivity (close to 1 mS cm^−1^), but also are endowed with better nonflammability and moisture-resistance than sulfide/halide SSEs^10,11^. The doped garnet-Li_7_La_3_Zr_2_O_12_ (LLZO) SSE shows the anode stability advantage over other oxide SSEs, such as NASICON-Li_1.3_Al_0.3_Ti_1.7_(PO_4_)_3_ and perovskite-Li_0.33_La_0.56_TiO_3_, due to the lacking of redox active elements (e.g., Ti, Ge)^12–14^.

Nevertheless, the interfacial problem between lithium metal and garnet electrolyte still exists in view of the potential passivation of ceramic grains by naturally formed Li_2_CO_3_, which results in poor wettability of LLZO surface by Li plating and large interfacial resistance^15,16^. In order to modify the interface contact, some methods have been attempted to eliminate Li_2_CO_3_ (e.g., by carbothermal reaction, high-temperature calcination, or acid treatment) or construct lithiophilic interlayer (e.g., by depositing alloyable film, decorating Li^+^-conductive polymer, pasting soft graphite, or two-dimensional MoS_2_)^17–25^. For the alloyable strategy, some expensive installations and refined manipulation for thin film deposition (e.g., atomic layer deposition and chemical vapor deposition) are usually required in order to achieve compact planar contact^21,26,27^. Although the facile addition of alloyable elements (e.g., Sn or graphite) into molten Li can also improve the wettability of anode on LLZO by tuning the surface tension and viscosity of molten lithium^28,29^, a high weight percent of blended alloy (e.g., blending 50% Sn or 70% graphite) is required for best wetting effect. In this case, the theoretical specific capacity of alloying anode would be significantly reduced compared with pristine Li anode.

Liquid metal (LM) gallium and gallium-based alloys have been widely used in the field of soft microfluidic electronics due to their low viscosity (2 mPa s), low toxicity, and negligible vapor pressure^30–32^. Although the high surface tension of pure LM makes it difficult to wet substrates under oxygen-lean condition, the self-formed Ga_2_O_3_ skin in an oxygenated environment can lead to the lowering of effective surface tension^32^. This oxide layer allows LM droplets to wet substrate surfaces and also stabilizes LM by preventing it from further oxidation. The Ga_2_O_3_ skin with a thickness of 0.5–3 nm behaves like an elastic membrane that can reform instantaneously when broken, therefore, enabling LM to be structurally self-stabilized under external multivariate conditions^33^. This particular wetting behavior of LM with intrinsic Ga_2_O_3_ skin makes it an available painting material on substrate surface or penetrating material in grain boundaries (GBs)^34,35^.

In this work, we propose a lithiophilic layer building strategy by brushing LM with excellent wetting behavior on garnet-based ceramic electrolyte surface to significantly reduce its interfacial resistance and assist the high reversibility of LMBs. This facile method does not require extra deposition equipment and exact (high) alloying content. The conformal oxide layer on LM enables a smooth brushing of LM paint on solid electrolyte surface, which can prevent garnet from further exposure to water and O_2_ in air. In addition, LM can infiltrate into the GBs of garnet to a certain depth, leading to a better interface transition effect. On the other hand, lithiated LM layer can provide extra Li^+^ transport channels and its high affinity with Li_2_CO_3_ enables the bypassing of charge transport from Li_2_CO_3_ passivation layer^36^. With the assistance of LM painting, the area-specific resistance (ASR) values of Li-garnet interface decrease to 5 Ω cm^2^ at 60 °C. The Li/Li symmetric cells can cycle for at least 9930 h with small overvoltage values.

## Results

### Wetting behavior of painted LM with naturally oxidized skin

**Fig. 1:**
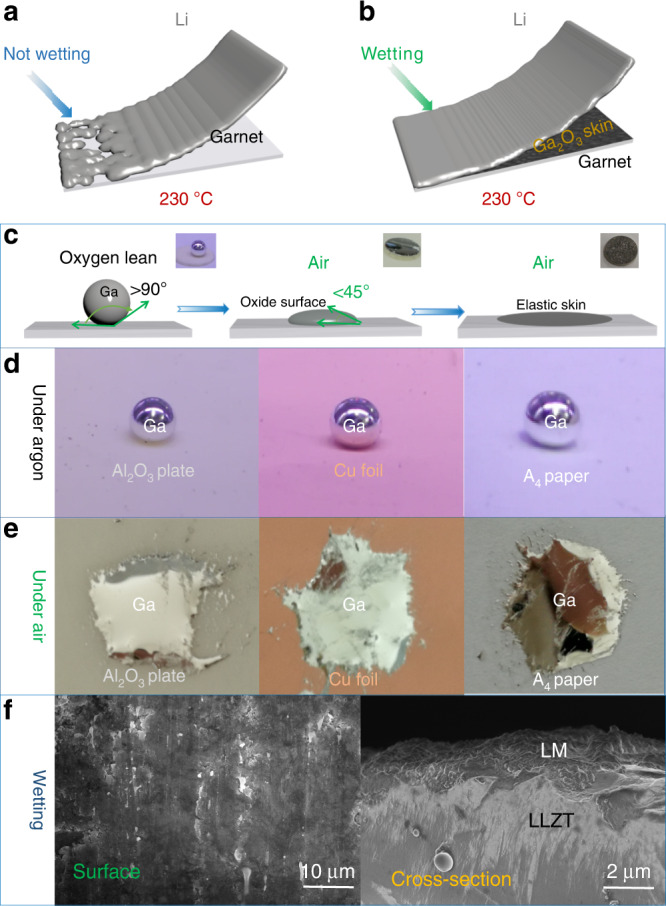
Wetting behavior of LM in air on garnet surface. Schematic of wetting behavior of molten lithium on the surfaces of (**a**) unmodified garnet and (**b**) liquid metal painted garnet at 230 °C. **c** Schematic of liquid metal on garnet surface in oxygen-lean atmosphere and air environment, as well as elastic gallium oxide residual on garnet surface. Insets: realistic wetting situation of liquid metal on garnet in corresponding environments. Wettability of liquid metal on different substrates (from left to right: Al_2_O_3_ plate, Cu foil, A_4_ paper) under (**d**) argon and (**e**) air environments. **f** Surface SEM image of liquid metal painted garnet. **g** Cross-section SEM image of LM-wetted garnet.

Herein, we choose Ta_2_O_5_ as doping agent for cubic garnet electrolyte in view of the structural stabilization and high ionic conductivity of Li_6.5_La_3_Zr_1.5_Ta_0.5_O_12_ (LLZT) electrolyte (Supplementary Figs. 1–3)^11^. The electrochemical impedance spectra (EIS) of Ag/garnet/Ag symmetric configuration were measured to obtain a RT ionic conductivity of LLZT of 4 × 10^−4^ S cm^−1^ with an activation energy of 0.36 eV (Supplementary Fig. 4). Pristine garnet electrolyte with a lithiophobic surface from insulating Li_2_CO_3_ causes poor wettability for lithium anode and therefore point contact between anode and electrolyte (Fig. 1a). There is a big gap that separates molten lithium from pristine garnet electrolyte surface (Supplementary Fig. 5). In contrast, with simple painting of gallium LM on garnet surface (marked as LM@LLZT) under air, a highly lithiophilic and conformal interlayer is expected to be constructed due to the elastic Ga_2_O_3_ skin coverage on LM inclusions (Fig. 1b). Under oxygen-lean condition (e.g., in vacuum or argon), LM has low viscosity and high surface tension, and exists in the form of nearly spherical shape, which is hard to wet garnet substrate and shows a large contact angle more than 90° (Fig. 1c). After LM is exposed to air to generate an oxide layer, significantly reduced surface tension and improved viscosity endow LM with excellent wettability and paint behavior^37^. After removing excess gallium metal, a dark film composed of residual elastic gallium oxide is well formed on garnet surface. The realistic photos at different stages are correspondingly provided as insets of Fig. 1c. To further certify the versatility of the unique painting behavior of LM, we dropped them on different substrates of Al_2_O_3_ plate, Cu foil and A_4_ paper (Fig. 1d, e). The excellent spreadability is also observed for all the substrates when LM is operated under air with the formation of self-passivating Ga_2_O_3_ film. This stable pasting behavior is caused by surface-dispersed Van der Waals force of gallium oxide, which is favorable for the gap or void healing at anode–electrolyte interface^38^. The scanning electron microscopy (SEM) images of LM@LLZT further certify the wetting behavior of surface-oxidized LM. Note that the Ga_2_O_3_ film enables a smooth and continuous coating on garnet grains (Fig. 1f and Supplementary Fig. 6), and the rough GBs are not discernable. From the view of cross-section images (Fig. 1g and Supplementary Fig. 7), LM with oxide skin totally wets the bumpiness zone and GBs at electrolyte surface without any gap exposure. Energy dispersive X-ray spectroscopy (EDS) mapping of LLZT proves the homogenous distribution of Ga element as consequence of favorable painting of LM (Supplementary Fig. 8).

**Fig. 2:**
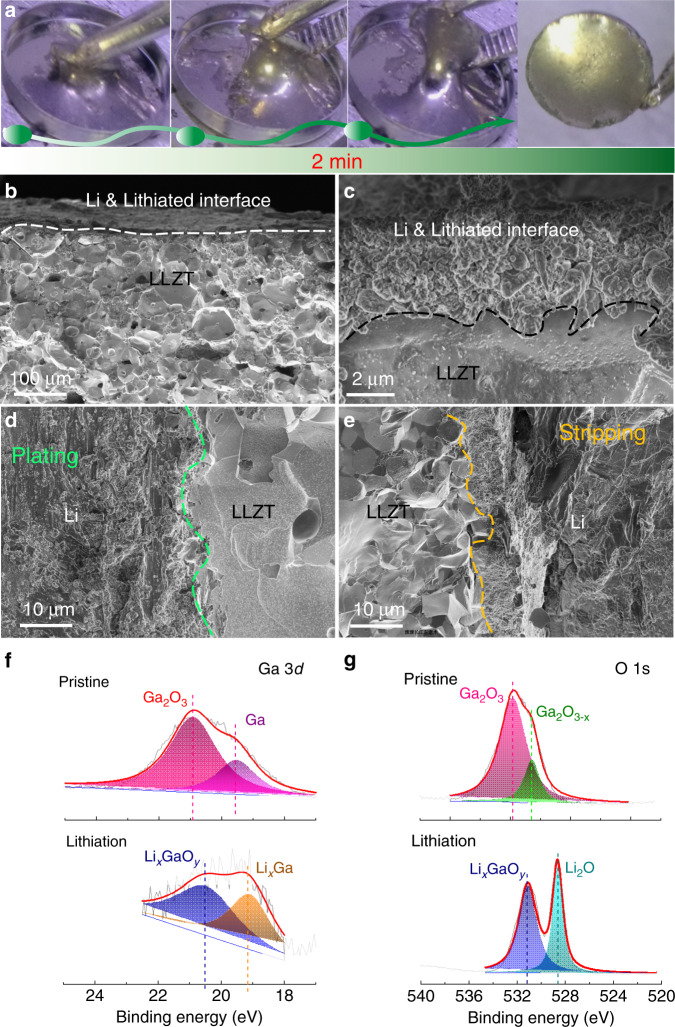
Improved wetting behavior of Li anode on LM-painted garnet. a Photos taken with time showing the lithiation process of LM-wetted garnet and photo of lithiated anode tightly adhered on garnet surface after 2 min. **b** SEM image of lithiated interface with liquid metal painting between Li and LLZT and (**c**) its enlarged SEM image to disclose the compact interface. Cross-section SEM images of interfaces between Li and liquid metal painted garnet after (**d**) Li plating and (**e**) stripping based on an area capacity of 1 mAh cm^−2^. XPS spectra of LM-decorated garnet surface exhibiting (**f**) Ga 3*d* and (**g**) O 1*s* spectra before and after lithiation.

When dipping LM@LLZT into molten lithium, fast lithiation can be achieved in merely 2 min (Fig. 2a), while molten lithium cannot wet the surface of pristine LLZT covered with an intrinsic lithiophobic skin (Supplementary Fig. 9)^17–20^. The garnet surface after lithiation shows a compact coverage of lithium with desired metallic luster. The improved wettability of Li metal toward LM-painted garnet is further confirmed in Supplementary Fig. 10. When a Li disc is heated at 200 °C, the molten Li spreads quickly and covers the entire surface of LM@LLZT within 5 min with the color turning from pristine dark (the color of gallium-oxide skin) to yellow (the color of Li). After lithiation, the smooth and continuous morphology of interlayer is well preserved (Fig. 2b, c). The excess Li and lithiated LM are elaborately blended without texture segregation phenomenon, indicating an excellent spatial compatiblity of LM interlayer with both anode and electrolyte. Even at a large capacity up to 1 mAh cm^−2^, the Li/LM@LLZT/Li symmetric cell can run stably (Supplementary Fig. 11). The newly formed interfaces after Li plating and stripping still show tight contact between Li and LLZT (Fig. 2d, e), benefiting from the high mobility of LM, which enables a prompt filling or wetting of pits or gaps left after Li stripping or deposition.

X-ray photoelectron spectroscopy (XPS) analysis was performed to characterize the surface components of LM@LLZT before and after lithiation (Fig. 2f, g). For pristine LM decoration, the Ga_2_O_3_ peak is found at 20.9 eV for Ga 3*d* as expected, and it is more intensive than that of Ga metal at 19.5 eV, which is conformally coated by the former^39,40^. For O 1*s* spectrum, the corresponding Ga_2_O_3_ peak is located at 531.9 eV^41^. The other should peak (with lower intensity) at 530.5 eV likely stems from the generation of Ga_2_O_3−*x*_ as a consequence of insufficient surface oxidation. After lithiation, Ga_2_O_3_ and Ga components are expected to convert into Li_*x*_Ga and Li_2_O products^42^. Therefore the Li_*x*_Ga (19.1 eV) and Li_2_O (528.5 eV) peaks become dominant in the Ga 3*d* and O 1*s* spectra, respectively^4^. Note that the undecomposed Li_*x*_GaO_*y*_ still remains from the appearance of peaks at 20.7 eV for Ga 3*d* and 531.1 eV for O 1*s*^40^. We also compare the corresponding XPS results before and after etching the surface for 10 s (Supplementary Figs 12 and 13). Note that the fraction of metallic Ga (from Ga 3*d* spectra) does not remarkably increase with the etching of Ga_2_O_3_ skin at outer surface, indicating that the oxidation depth is not very shallow. The increased fraction of Ga_2_O_3−*x*_ (compared with Ga_2_O_3_, from O 1*s* spectra) on etched surface agrees with the attenuation or reduction of Ga_2_O_3_ during etching process. After lithiation (Supplementary Fig. 13), the Li_*x*_GaO_*y*_ fraction increases (compared with Li_2_O, from O 1*s* spectra) in the etched sample, indicating the lithiated conversion reaction progresses more sufficiently at the outer region than at the inner region. This phenomenon agrees with the increased fraction of Li_*x*_GaO_*y*_ component (compared with Li_*x*_Ga) from Ga 3*d* spectra. The slight inhomogeneity of lithiation process does not influence the Li-ion transport across LM coating and its oxidation skin. The phase assignment of lithiated LM is further confirmed by X-ray diffraction (XRD) pattern (Supplementary Fig. 14a). The Li_*x*_Ga alloy contains the phases of Li_2_Ga and Li_3_Ga_2_, and Li_*x*_GaO_*y*_ is composed of LiGaO_2_, Li_5_GaO_4_, and LiGa_5_O_8_ phases. The existence of these phases is consistent with the Li-Ga-O ternary phase diagram (Supplementary Fig. 14b).

### Li_2_CO_3_-affiliative mechanism via LM painting

**Fig. 3:**
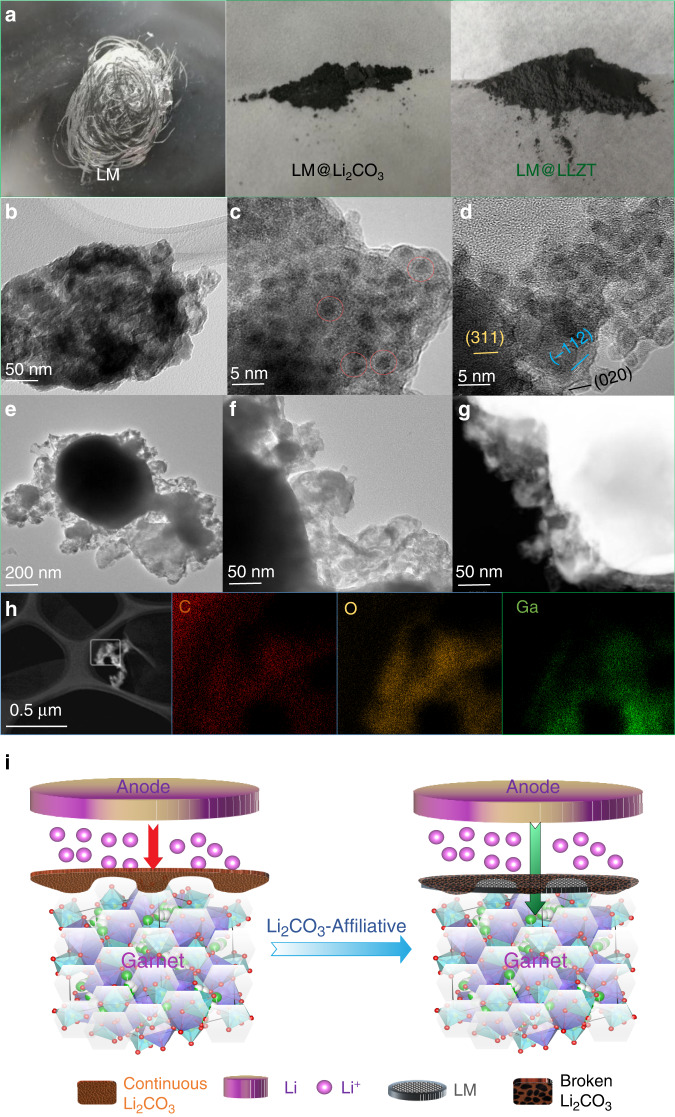
Confirmation of Li_2_CO_3_-affiliative mechanism via blending with LM. a Photos of liquid gallium after grinding in air environment and its mixtures with Li_2_CO_3_ powder and cubic garnet powder. **b** TEM image of Li_2_CO_3_ powder with the embedment of liquid metal domains. **c**, **d** HRTEM of well-mixed LM and Li_2_CO_3_ nanodomains in different zones. **e** TEM image of interfacial contact between excess liquid metal and Li_2_CO_3_ and (**f**) its magnified region to disclose the affiliative interface of LM-Li_2_CO_3_. **g** HADDF image of interfacial situation between LM sphere and Li_2_CO_3_ moieties. Liquid metal including in its oxide skin is bright while the rest part is Li_2_CO_3_ powder. **h** STEM and corresponding EDS mapping images of C, O, Ga elements. **i** Schematic comparison of Li-ion interface transfer across continuous Li_2_CO_3_ layer and across broken Li_2_CO_3_ network wetted with liquid metal.

Note that the good contact between LLZT and LM can be obtained even without intentional removal of the so-called passivated Li_2_CO_3_. There is a speculation that alloyable element (e.g., Zn) may react with Li_2_CO_3_ to create more Li vacancies in passivation layer for faster Li-ion transport at the interface zone^43^. However there is no strong evidence to support this prediction. Herein, we propose a Li_2_CO_3_-affiliative mechanism in which Li_2_CO_3_ on garnet surface is wetted and downsized by LM drops, and it is torn into Li_2_CO_3_ nanodomains by surrounding LM nanoparticles with similar nanoscale. The superior wettability can guarantee a homogeneous mixing of Li_2_CO_3_ and LM nanodomains. Therefore the separated Li_2_CO_3_ grains cannot form continuous passivation layer to retard Li-ion transport. Instead, the well-dispersed LM grains can serve as ion wires after lithiation to construct the alternative ion channels especially when they penetrate into continuous conductive network. To prove our hypothesis, we ground LM with Li_2_CO_3_ or garnet powder (Fig. 3a). Under the mechanical force during the mixing process in air, the shiny LM is pulverized into much smaller particles that blend with white Li_2_CO_3_ or light-yellow garnet powder. The resultant darkening stems from the higher-fraction oxidized regions of tearing LM grains by air or oxide powder. In contrast, the pristine LM still maintains its metal luster even after grinding in air in view of the self-limiting surface oxidation. These phenomena indicate the facile dispersion capability of LM when contacting with carbonate or ceramic powder. The strong Van der Waals’ force of self-formed gallium-oxide skin is responsible for its coverage on powder grains^38^. The XRD pattern of the mixture of LM and Li_2_CO_3_ (LM@Li_2_CO_3_) does not display the evident peaks ascribed to LM (Supplementary Fig. 15), further indicating the attenuation of LM after dispersing and downsizing. The enrichment of gallium oxide does not cause the appearance of excess diffraction peaks due to its relatively poor crystallinity^37,44^. The pronounced peaks of Li_2_CO_3_ are still preserved after mixing with LM, considering the mechanical robustness of well-crystallized Li_2_CO_3_. Similarly, the mixing of LM with LLZT (LM@LLZT) also causes the weakening and elimination of LM diffraction peaks (Supplementary Fig. 16). The cubic phase structure of LLZT is not destroyed after mixing with LM.

To further explore this fascinating wetting phenomenon of LM with Li_2_CO_3_ powder, transmission electron microscopy (TEM) was resorted to. Fig. 3b discloses the excellent mutual miscibility between LM and Li_2_CO_3_ nanodomains, and there is no serious phase segregation observed. Under high resolution (Fig. 3c, d), we clearly observe the homogenous distribution of LM and Li_2_CO_3_ nanoparticles with comparable sizes as small as 3–5 nm. The LM particles are discerned from the darker spots, while the Li_2_CO_3_ ones are the crystallized domains with typical lattice fringes corresponding to (311), (020), (−112) planes with *d*-spacings of 0.187, 0.249, 0.262 nm, respectively (Supplementary Fig. 17). In view of the actual interface with excess LM modification compared with the amount of naturally formed Li_2_CO_3_, we also intentionally blended higher-fraction LM with Li_2_CO_3_ to see the microscopic distribution of mixture. As shown in Fig. 3e–g, some big drop-like spheres of LM are still residual without undergoing the fragmentation due to the insufficiency of carbonate. However their oxidation surface still has good affiliative ability toward surrounding Li_2_CO_3_, leading to the appearance of Li_2_CO_3_ moieties anchored on LM sphere surface. A part of Ga_2_O_3_ skins are likely peeled off and enter into the Li_2_CO_3_ network to form the mutual mixture. This would result in the interaction reinforcement at Li_2_CO_3_-LM interface as indicated from the firm attachment of LM to the Li_2_CO_3_-covered LLZT surface even after Li plating and stripping. The scanning transmission electron microscopy (STEM) image and corresponding EDS mapping of C, O, Ga (Fig. 3h) disclose the similar spatial distributions of carbonate and LM components, further confirming the uniform blending of both the phases after grinding. This Li_2_CO_3_-affiliative mechanism mitigates the passivation effect and reinforces the interconnection of conductive interface (Fig. 3i). The penetration of LM conductive network along with the breaking up of carbonate passivation layer provides an alternative pathway for facile Li-ion transport across Li-LLZT interface without the requirement on removing the passivation layer by harsh conditions^17,18^.

Raman spectra of garnet pellet, gallium mixed Li_2_CO_3_, and gallium mixed garnet powders are represented in Supplementary Fig. 18. The garnet pellet shows the typical characteristic peaks roughly located at 243, 376, 645, and 728 cm^−1^ (corresponding to instinct signals of cubic LLZT, the former two peaks being ascribed to the Li–O bonding), apart from the strong CO_3_^2−^ vibration peak at 1090 cm^−1^(ref.^17^). After mixing with LM, the characteristic peaks of garnet are covered, while the Li_2_CO_3_ peak is still maintained. This phenomenon indicates a potential interaction between LM (or its oxide skin) and LLTO surface. Compared with the Raman spectrum of LM mixed Li_2_CO_3_, we deduce that the broad peak located at 700 cm^−1^ should stem from the vibration of Ga–O bonding in both the mixture samples^45^. Note that the blending of LM cannot reduce or decompose the Li_2_CO_3_ component, agreeing with the XRD results mentioned above.

### Solid-state cells benefitting from superior LM wettability

**Fig. 4:**
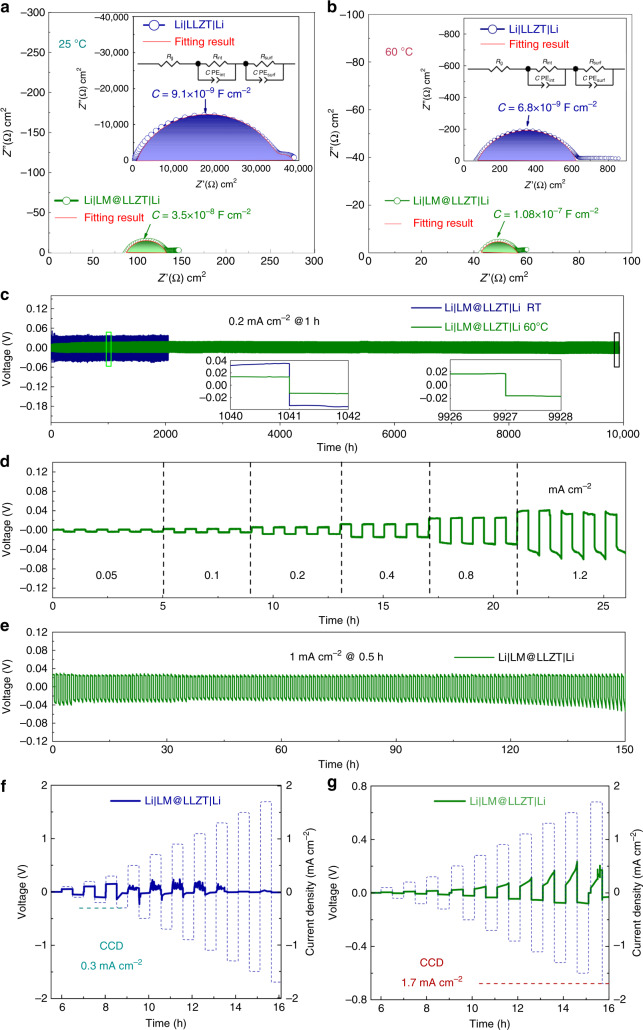
Electrochemistry of Li/Li symmetric cells based on LM-wetted garnet. Electrochemical impedance spectra of Li|LM@LLZT|Li symmetric cells at (**a**) room temperature and (**b**) 60 °C. Insets: corresponding impedance spectra of Li|LLZT|Li symmetric cells without any modification. **c** Long-term performance of Li|LM@LLZT|Li symmetric cells at 0.2 mA cm^−2^ at room temperature and 60 °C. Insets: corresponding voltage profiles at different cycling stages. **d** Rate performance of Li|LM@LLZT|Li symmetric cell from 0.05 to 1.2 mA cm^−2^. **e** Li plating-stripping performance Li|LM@LLZT|Li symmetric cell at a high current density of 1 mA cm^−2^. Critical current density measurement of Li/Li symmetric cells based on (**f**) unmodified garnet (with 10 uL of liquid electrolyte adding) and based on (**g**) liquid metal painted garnet.

To characterize the electrochemical benefit from this strategy, lithium symmetric cells were assembled with the LM-painted LLZT (marked as Li|LM@LLZT|Li) and unmodified garnet (marked as Li|LLZT|Li) as solid electrolytes, respectively. The EIS of Li/Li symmetric cells was performed to evaluate the interfacial situation. The interfacial ASR values of symmetric cells are significantly decreased from 1.75 × 10^4^ to 19.5 Ω cm^2^ at RT, and from 272 to 5 Ω cm^2^ at 60 °C after LM painting on both the sides of garnet (Fig. 4a, b)^43^. The ASR values are estimated based on the equivalent circuit in the insets of Fig. 4a, b, which shows good fitting effect^46^. Therein *R*_g_, *R*_int_, and *R*_surf_ denote the ASRs for garnet, interface transfer, and surface reaction, respectively. CPE_int_ and CPE_surf_ denote the constant phase elements paralleled with *R*_int_ and *R*_surf_, respectively. The corresponding characteristic time constants (*τ*) and capacitance values (*C*) are listed in Supplementary Table 1. *C* and *τ* can be expressed by (*R*^1−*n*^CPE)^1/*n*^ and RC, where *n* is CPE exponent. The capacitance values referring to interfacial transport are in the range of 10^−9^–10^−7^ F cm^−2^, while those referring to surface reaction are in the range of 10^−7^–10^−5^ F cm^−2^. The discrepancy of capacitance range depending on different process is in accordance with the precious report by Irvine et al.^47^ After LM painting, the relaxation time is shortened no matter for the interfacial or surface process, also indicating an improved electrochemical kinetics. Both the ASR values of Li|LM@LLZT|Li cell at RT and 60 °C are smaller than most of reported values even if the garnet pellets we used have been intentionally exposed to air for several days (Supplementary Table 2). These comparisons demonstrate the superiority of this facile painting strategy.

The symmetric Li|LM@LLZT|Li cells can achieve an ultralong Li plating/stripping cycling for at least 9930 h (4965 cycles) at 60 °C with an ultra-small overpotential from −10 to 10 mV, as well as from −33 to 33 mV at RT at a current density of 0.2 mA cm^−2^ based on an areal capacity of 0.2 mAh cm^−2^ (Fig. 4c). The symmetric cell at RT can be steadily cycled for at least 2000 h. Note that the Li plating and stripping curves of symmetric cells are quite plat and smooth during the early cycling and after long-term cycling (insets of Fig. 4c), indicating the elimination of nucleation overpotential as a consequence of facile charge transport across lithiated LM. In all-solid-state architecture, there is no extra accumulation of solid electrolyte interface from side reaction to cause larger nucleation resistance and overpotential. The modification of impedance and cycling performance also benefits from the prior Li melting step (230 °C for 5 min) for better interface contact. Note that the symmetric cell even without Li melting pretreatment can also cycle for a long time over 750 h, but at a cost of unstable polarization activation process during early cycling (Supplementary Fig. 19). After activation, the overpotential is quite stable and is still small (from −20 to 20 mV) at 60 °C, benefiting from the construction of mixed conductive network induced by electrochemical lithiation of LM interlayer. In contrast, the symmetric cell with unmodified garnet reaches to the short circuit stage quickly after merely several hours (Supplementary Fig. 20). Before short circuit, the overpotential is also large and asymmetric between plating and stripping (with a gap of ~1.3 V) even at a smaller current density of 0.1 mA cm^−2^. The appearance of tip phenomenon in voltage profiles is caused by the poor interface contact^1,2^. For the LM-modified symmetric cell, stable rate performance is recorded with the increase of current density from 0.05 to 1.2 mA cm^−2^ as shown in Fig. 4d. The corresponding voltage profiles do not undergo serious degradation, and they are roughly flat even at much higher current density exceeding 1 mA cm^−2^. Even when reaching to 1 mA cm^−2^, the overpotential is still controlled between −28 and 28 mV (Fig. 4e). This low plateau overpotential does not increase remarkably and can last for at least 150 h during the following cycling. A high critical current density (CCD, defined as the highest applied current density that the solid-state electrolyte can endure the lithium dendrite penetration) value up to 1.7 mA cm^−2^ can be reached for the LM improved symmetric cell (Fig. 4g), while the CCD value for unmodified cell is much smaller (0.3 mA cm^−2^) even with the assistance of LE wetting (Fig. 4f). This high CCD value in this work is superior to most of the literature reports (e.g., 0.5 mA cm^−2^ for Sn film alloying, 0.8 mA cm^−2^ for H_3_PO_4_ modification, 1 mA cm^−2^ for Li-graphite anode), indicating an excellent defense capability of LM interlayer against Li dendrite growth^20,29,48^. As suggested by Flatscher et al.^46^, CCD is highly related to the conditions of interface wetting, pressure, and temperature. The obtained high CCD value also confirms the perfect Li wetting and agrees with the significantly reduced ASR values at the fixed conditions without externally applied pressure and high temperature.

**Fig. 5:**
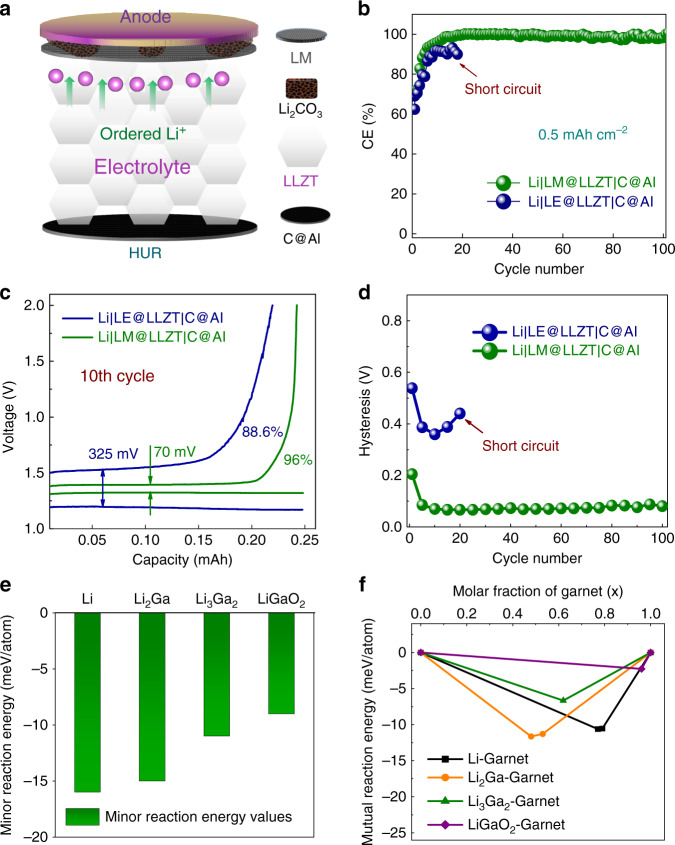
Electrochemistry of asymmetric cells and interface stability evaluation. **a** Schematic of ordered Li^+^ flux across LM-modified interface, leading to high utilization ratio (HUR) of Li anode. **b** CE values of Li|LM@LLZT|C@Al and Li|LE-LLZT|C@Al asymmetric cells with carbon-coated allium foil as counter electrode as a function of cycle number at 0.1 mA cm^−2^. **c** Voltage profiles of both the asymmetric cells at the 10th cycle. **d** Comparison of voltage hysteresis evolution of both the asymmetric cells within 100 cycles. Calculated (**e**) minor reaction energies and (**f**) mutual reaction energies between main interface phases (after lithiation) and garnet solid electrolyte.

Since the symmetric cell architecture employs excess Li, it is not suitable to evaluate the accurate utilization ratio of anode as well as accumulation degree of dendritic Li or dead Li in GBs. To further explore the dendrite suppression effect modulated by LM painting, we also performed the asymmetric cells, which were not often used to estimate the Li plating behavior. Herein the carbon-coated Al foil (denoted as C@Al) is used as the nonlithium electrode. The asymmetric cell with solid electrolyte painted by LM is denoted as Li|LM@LLZT|C@Al, while the control cell with extra LE dropped on the anode side of garnet is denoted as Li|LE@LLZT|C@Al. Benefiting from the excellent wettability of LM and its dissipation effect on passive Li_2_CO_3_, the lithiated interphase layer allows a uniform Li^+^ flux from bulk electrolyte to anode, thus resulting in a high utilization ratio of Li (Fig. 5a). In contrast, for control cell, the dendrites are expected to initiate at interface and grow inside garnet due to the uneven Li^+^ flux frustrated by continuous Li_2_CO_3_, causing a low utilization ratio of Li even with LE addition (Supplementary Fig. 21). The coulombic efficiencies (CEs) for Li|LM@LLZT|C@Al cell are well stabilized at the high values close to 100% for at least 100 cycles after early activation (Fig. 5b), while the Li|LE@LLZT|C@Al cell can only run for <20 cycles with a smaller CE value of ~90% before failure. The former cell has a much smaller voltage hysteresis (e.g., 70 mV in the 10th cycle) than that (325 mV) for the latter cell in the same cycling stages (Fig. 5c). The voltage hysteresis after LM painting is preserved at a low value without serious fluctuation during the whole cycling process (Fig. 5d). For unmodified cell, the voltage hysteresis increases rapidly after ten cycles. These results further confirm the improved reversibility and kinetics of Li plating through the stable LM interlayer, leading to the alleviations of active Li roughening and dead Li formation.

Interface reactions between lithiated LM components and LLZT were also studied (based on first-principle computations)^49,50^. The interface is considered as a pseudo-binary structure of lithiated interphase and garnet and the most stable phase equilibria based on thermodynamic driving forces is determined. Lithiated interphase (e.g., main components Li_2_Ga, Li_3_Ga_2_, and LiGaO_2_) shows better chemical stability with garnet (than Li metal) with a minor reaction energy more than −15 meV/atom (Fig. 5e), which is higher than that of Li-LLZT and much higher than previously reported values (e.g., −40 to −90 meV/atom for Si-garnet, −24.78 to −62 meV/atom for Al-garnet, −20 to −100 meV/atom for Li_*x*_C-garnet)^21,29,51^. Therefore the side reaction between lithiated LM and LLZT is greatly mitigated. The mutual reaction energy shows the similar trend (Fig. 5f) and this moderate mutual reaction might assist the wetting of LM-alloyed anode with garnet surface (especially for Li_2_Ga component). Since the wettability of molten lithium on Li_2_CO_3_-free garnet is not bad^52^, the much more negative mutual reaction energy (than −10.54 to −10.61 meV/atom for Li-garnet in Supplementary Table 3a) may be not necessary. The higher minor reaction energy of lithiated interphases than pure Li would not cause serious interface passivation and be favorable for the endurance in terms of long cycling, high current density, and large area capacity of symmetric cells. We also considered the potentiality of reaction between other possible component (e.g., LiGa, Li_2_Ga_7_, Li_5_Ga_4_ undetectable from XRD of lithiated LM) or trace phase (LiGa_5_O_8_) and garnet electrolyte. There is no probable reaction occurring between LiGa or Li_2_Ga_7_ and ceramic, while Li_5_Ga_4_ has a low reactivity based on its reaction energy of −6 meV/atom with garnet (Supplementary Table 3d), which is higher than the decomposition energy of garnet (−7 meV/atom). The trace LiGa_5_O_8_ phase shows the more negative reaction energy values (Supplementary Table 3f) comparable with those for Al and graphite^29,51^, and its possible reaction products are still stable Li_5_GaO_4_ and LiGaO_2_ phases (no reaction between Li_5_GaO_4_ and garnet). Therefore the Li-Ga-O interphases would not passivate the interface with LLZT.

**Fig. 6:**
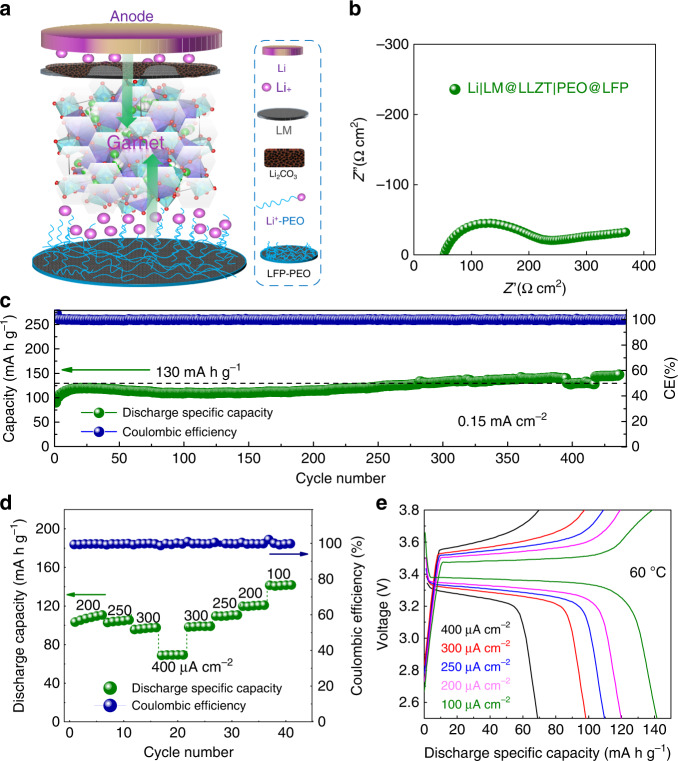
Cycling performance of solid-state cells based on LiFePO_4_ (LFP). **a** Structure schematic of Li|LM@LLZT|PEO@LFP solid-state cell. **b** EIS spectrum of fresh cell before cycling. **c** Cycling performance of solid-state cell at 0.15 mA cm^−2^. **d** Rate performance of solid-state cell at the current densities from 0.1 to 0.4 mA cm^−2^. **e** Voltage profiles of solid-state cell at different current densities from 0.1 to 0.4 mA cm^−2^.

LM-painted garnet is expected to drive the solid-state LMBs with better kinetic performance. The Li|LM@LLZT|PEO@LiFePO_4_ cell contains LiFePO_4_ cathode composited with polyethylene oxide (PEO) and lithium bis-(trifluoromethanesulfonyl) imide (LiTFSI) salt as Li-ion wire (Fig. 6a). The interfacial resistance of full cell including both the contributions of anode and cathode interfaces is ~150 Ω cm^2^ (Fig. 6b). This resistance value is dominated by the contribution of LLZT-cathode interface in view of the small resistance (5 Ω cm^2^) of Li-LM@LLZT interface. This cathode interface resistance (143 Ω cm^2^) is not high and comparable to those in previous reports based on polymer decorated cathodes^17,23,53^. This solid-state cell enables an ultralong cycling performance with a highly stable capacity around 130 mAh g^−1^ after 440 cycles at 0.15 mA cm^−2^ under 60 °C (Fig. 6c). The corresponding CEs are quite stable and close to 100%. This cell displays good rate performance with capacities of 110, 100, 70 mAh g^−1^ at 0.25, 0.3, 0.4 mA cm^−2^, respectively (Fig. 6d, e). The high CEs are not influenced by the increase of current density. The hard Li-LLZT modified interface is responsible for the high cycling endurance. Otherwise, the soft interface (e.g., Li-PEO) is prone to cause overcharge phenomenon after few cycles (Supplementary Fig. 22) as a consequence of inferior Li dendrite suppression effect (Supplementary Fig. 23). Even under RT, the solid-state cell can also be successfully cycled (Supplementary Fig. 24). Fully dried garnet-based cell enables the good reaction kinetics by this Li_2_CO_3_-affiliative strategy without the requirement to remove the intrinsic passivation layer.

## Discussion

The advantage of LM painting decoration lies in the high affinity between LM-oxide skin and Li_2_CO_3_, which enables the disconnection of Li_2_CO_3_ network into Li_2_CO_3_ fragments separated by LM-oxide nanodomains. Therefore Li-ions can bypass the insulating Li_2_CO_3_ moieties and instead transfer along the lithiated LM and oxide domains at the interlayer zone. This dissipation effect of Li_2_CO_3_ is expected to become more pronounced after lithiation as indicated by the peak evolution of Li_2_CO_3_ XPS spectra (Supplementary Figs. 25 and 26). For the pristine LM@LLZT surface, the Li_2_CO_3_ peak is weakened and the La peaks become pronounced after etching process. This is an expected result because the upper Li_2_CO_3_ coverage is attenuated and meantime more LLZT surfaces are exposed during etching. In contrast, for the lithiated LM@LLZT sample, the Li_2_CO_3_ peak in turn becomes stronger after etching. Since the lithiation treatment thickens the LM interlayer, the La signals are not evident even after etching. The concentration increase of Li_2_CO_3_ in the lithiated interlayer is likely caused by the extrusion of Li_2_CO_3_ fragments from the monolithic Li_2_CO_3_ beneath in view of the volume expansion during lithiating LM. The Li_2_CO_3_ domains can even be pushed to the top surface of lithiated LM@LLZT. This crowding-out effect is expected to further reinforce the dispersal of Li_2_CO_3_ and promote the Li-ion flux at interlayer. The microstructure and crystallinity evolution of lithiated gallium oxide likely influences its interaction with Li_2_CO_3_ and should be responsible for the observation of Li_2_CO_3_ peak position shifting. The poor crystallinity of oxide skin endows it with better surface attachment ability^38^. Therefore some LM domains with small size can penetrate into the GBs of LLZT after breaking Li_2_CO_3_ passivation layer. When lithiation occurs, the prepenetrated LM of high mobility can shuttle back to the interlayer based on the Li-LM alloying force. This shuttling process can further delaminate and fragment the Li_2_CO_3_ network, agreeing with the XPS result afore-mentioned. In brief, both the embrittlement and penetration effects of LM enable a superior manipulation from continuous Li_2_CO_3_ layer to its nanoscale fragments^35^.

The chemical Li diffusion coefficient (*D*_Li_) for Li–Ga alloys has a high value around 10^−8^–10^−6^ cm^2^ s^−1^ at normal temperature (24–30 °C), which is comparable to those of Li–In and Li–Al alloys^54^. Both the high Li flux in Li–Ga interlayer and improved interface contact are responsible for the driving of high current density. The high Li flux in Li–Ga alloy stems from the high solubility of Li in Ga metal with the existence of multiply intermetallic phases of Li-rich alloys with kinetically favorable conversion. The intimate interface contact can heal the morphological defects and homogenize the lithium growth^55^. Therefore the high Li flux path can be well connected from solid electrolyte to electrode. The high CE values and small voltage hysteresis of Li–Ga modified asymmetric cell can act as evidences for the high utilization of Li and fast interfacial kinetics (Fig. 5b, c).

In summary, we propose a Li_2_CO_3_-affiliative mechanism to modulate garnet electrolyte interface by facilely painting LM coating. This strategy enables a superior wettability of LM with naturally oxidized skin toward both Li metal and LLZT under oxygenated environment. It avoids the requirement on the removal of Li_2_CO_3_ passivation layer, which can be delaminated and fragmented by LM penetration. The lithiated LM nanodomains can construct alternative Li-ion transfer route at Li-LLZT interface. Benefiting from this Li_2_CO_3_-affiliative mechanism, the interfacial ASR between Li and garnet is as small as 5 Ω cm^2^. The symmetric cell of Li|LM@LLZT|Li can cycle for ultralong 9930 h with a small overpotential not more than 12 mV. Even at a high current density of 1 mA cm^−2^, the overpotential is still <30 mV. This work provides a scalable way to significantly improve the interface performance of garnet electrolyte even with exposure to air for several days. The modification direction of designing Li_2_CO_3_-affiliative interlayer with dilution and embrittlement of Li_2_CO_3_ nanodomains should be emphasized for further development of garnet-based solid-state batteries.

## Methods

### Synthesis of solid garnet electrolyte

The Li_2_CO_3_, ZrO_2_, La_2_O_3_, and Ta_2_O_3_ materials (Shanghai Aladdin Bio-Chem Technology Co., Ltd) with certain molar ratio were used as precursors to prepare garnet Li_6.5_La_3_Zr_1.5_Ta_0.5_O_12_ (LLZT) by solid-state sintered technology. Fifteen percent excess Li_2_CO_3_ was added to offset the volatile loss of lithium in sintering process. La_2_O_3_ powder was calcined at 900 °C for 12 h to remove crystal water before sintering. These precursor materials were firstly mixed together with absolute ethyl alcohol and then ball-milled for 12 h at 230 r min^−1^. The mixed dry powder was sintered at 900 °C for 12 h to achieve tetragonal LLZT. The tetragonal garnet acquired in the previous step was broken into pieces and ball-milled for 24 h. Then the dry powder was pressed into pellet and sintered at 1250 °C for 1 h and 1150 °C for 6 h to obtain the final cubic garnet electrolyte. All of sintered garnet pellets were exposed in air for 7 days before use. The surface area and thickness of ceramic pellets were fixed at about 0.5 cm^2^ and 0.8 mm, respectively. The density value of used pellets ranges from 90 to 92%.

### Liquid metal painting and cell fabrication

LM (Shanghai Aladdin Bio-Chem Technology Co., Ltd) was painted on LLZT surface at 35 °C with a brush until the whole garnet surface is wetted and coated by gallium with a dark Ga_2_O_3_ skin. The excess LM was carefully removed with a brush. This procedure was performed under normal air atmosphere. For interfacial resistance testing, the coin symmetric cell was assembled by melting lithium on LM-painted garnet for 5 min at 230 °C with Ni foam as current collector. During the assembly of asymmetric cell, 10 μL of commercial carbonated electrolyte (1 M LiPF_6_/EC:DEC, Aladdin) was dropped on the non-Li collector (carbon-coated Al foil, denoted as C@Al, purchased from Hefei Kejing Co., Ltd) for interface wetting. This C@Al electrode was then attached to garnet electrolyte, which was attached by Li anode at the other side. For the asymmetric cell based on unpainted garnet, the Li anode side was also wetted by 10 μL of LE, apart from the already wetted C@Al electrode side. To fabricate composite cathode, LiFePO_4_ powder (100 mg) was mixed with carbon black (60 mg), PEO (Aladdin) (246 mg) and LiTFSI (Sigma-Aldrich) salt (80 mg). The electrode slurry was prepared by mixing this mixture powder with acetonitrile for 12 h and then was pasted on clean carbon-coated Al foil and dried at 60 °C under vacuum overnight. The composite cathode has an active species loading of 2 mg cm^−2^. For polymer electrolyte fabrication, LiTFSI salt was firstly mixed with PEO in acetonitrile solution based on a molar ratio of [EO]:Li^+^ of 15:1, which was then stirred for 8 h. Then the solvent was evaporated and the polymer film was dried in vacuum.

### Electrochemical measurement

To measure the ionic conductivity of sintered LLZT electrolyte, Ag paste which can endure high temperature was coated on ceramics and calcined at 150 °C and then 800 °C for 10 min, respectively, to remove the organic component and ensure a tight contact. Then the EIS of Li^+^ blocking cell (Ag/LLZT/Ag) was measured by using a Solartron frequency analyzer (1260–1296) in a frequency range from 10^−2^ to 5 × 10^6^ Hz with an AC amplitude of 10 mV. The EIS spectra of symmetric cells were also measured to estimate the interface ASR with an applied frequency range from 10 MHz to 1 Hz at RT or 60 °C. For the electrochemical performance of symmetric cell, cycling process was performed at a current density of 0.2 or 1 mA cm^−2^ with a fixed plating/stripping interval of 1 or 0.5 h, respectively. The rate performance was performed under the current densities ranging from 0.05 to 1.2 mA cm^−2^ with a fixed plating/stripping interval of 0.5 h. For the CCD measurement, the stepped current density test protocol from 0.1 mA cm^−2^ (1 h per cycle, 0.2 mA cm^−2^ per step) was employed. The asymmetric cell was discharged at a current density of 0.1 mA cm^−2^ and then was charged to 1.0 V after 5-h deposition of Li. The solid-state full cells were measured at a current density of 0.15 mA cm^−2^ or at a changed rate ranging from 0.1 mA cm^−2^ to 0.4 mA cm^−2^ in a voltage range of 2.5–3.8 V. All the assembled coin cells were tested on a LAND-CT2001A Battery Test System.

### Physical characterization

SEM (Magellan 400 L, FEI) and EDS mapping were used to analyze the morphology and component distribution of garnet electrolyte and its interface. For the morphology observation of electrolyte surface and cross-section, LLZT pellet was smashed to obtain the samples. To prepare the cross-section interface, the Li|LLZT|Li symmetric cell was firstly assembled under a pressure of 50 MPa. Under this pressure, the Li|LLZT|Li trilayer structure was compacted but it is prone to be smashed. Then the smashed cell was disassembled to choose the suitable fragment for cross-section morphology characterization. The prior assembly of symmetric cell guarantees a relatively uniform pressure on the whole interface. Both the pristine and modified interface samples were prepared in this way in order to achieve a comparable result. XRD (BrukerD8 ADVANCE, Cu Kα source) was used to characterize the phase constitutions of sintered garnet and LM-painted electrolyte, as well as interface components after lithiation. Air-exposed garnet and LM-painted LLZT pellet were directly tested, while the interface component sample was firstly alloyed with lithium and then the interface was peeled off from garnet for XRD test. All the samples involved with lithium metal were fabricated in Ar-filled glove-box and sealed in a box when transferred to the testing chamber. The TEM with energy dispersive spectroscopy (JSM-6700F, JEOL) was operated at 200 kV to characterize the interfacial structure and components of LM mixed Li_2_CO_3_ powder. The sample used in TEM was distributed in ethanol under sonication and then was deposited on a Cu wire mesh. Raman spectra for the garnet powder, LM mixed garnet powder and LM mixed Li_2_CO_3_ powder were collected by a thermal dispersive spectrometer excited by a laser with the wavelength of 532 nm and the power of 10 mW. To explore the interface components of LM@LLZT before and after lithiation, XPS measurement (ESCAlab-250) with an Al anode source was also performed. Sample with fresh surface of LM@LLZT was fabricated by simply painting LM on air-exposed garnet surface, while the lithiated sample was firstly lithiated by melting a lithium disc of 0.3 cm^2^ for 5 min and then the surface-lithiated garnet was peeled from Li disc.

## Supplementary information

Supplementary Information

## Data Availability

The data that support the findings of this study are available from the authors on reasonable request, see author contributions for specific data sets.
