# Supplementary material for: Li2CO3-affiliative mechanism for air-accessible interface engineering of garnet electrolyte via facile liquid metal painting
Source: Nat Commun. 2020 Jul 24;11:3716. doi: 10.1038/s41467-020-17493-x (PMC7382479; doi:10.1038/s41467-020-17493-x)
Supplement: Supplementary file 1 — Supplementary Information [file 41467_2020_17493_MOESM1_ESM.pdf]

Supplementary Information for

Li_2CO_3-Affiliative Mechanism for Air-Accessible Interface Engineering of Garnet Electrolyte via Facile Liquid Metal Painting

Junwei Meng[a,b], Yang Zhang[a,b], Xuejun Zhou[a], Meng Lei[a], Chilin Li[a,b,*]

[a] State Key Laboratory of High Performance Ceramics and Superfine Microstructure, Shanghai Institute of Ceramics, Chinese Academy of Sciences, 585 He Shuo Road, Shanghai 201899, China. Email: chilinli@mail.sic.ac.cn

[b] Center of Materials Science and Optoelectronics Engineering, University of Chinese Academy of Sciences, Beijing 100049, China.

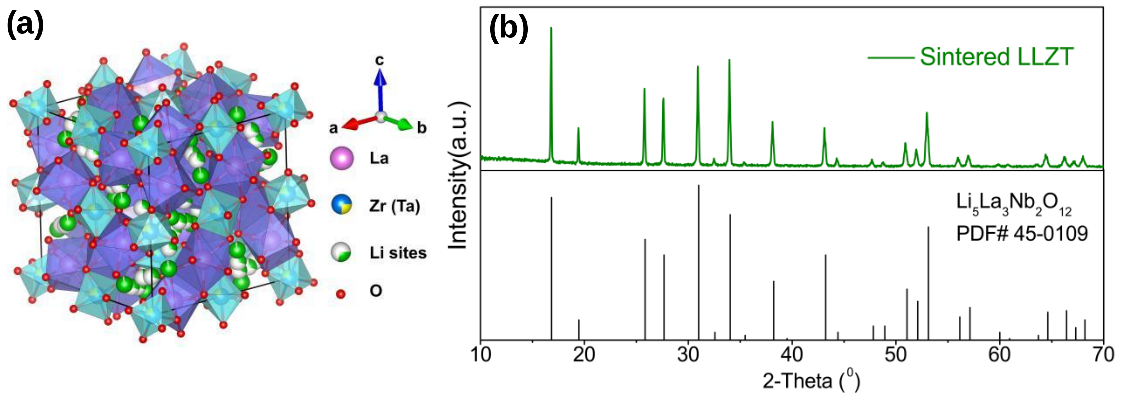

Supplementary Figure 1. (a) Cubic structure of $Li_{6.5}La_3Zr_{1.5}Ta_{0.5}O_{12}$ (LLZT) and (b) its X-ray diffraction (XRD) pattern. XRD demonstrates that the sintered fresh sample has a cubic crystal structure of garnet phase according to the pattern of $Li_5La_3Nb_2O_{12}$ (PDF#45-0109).

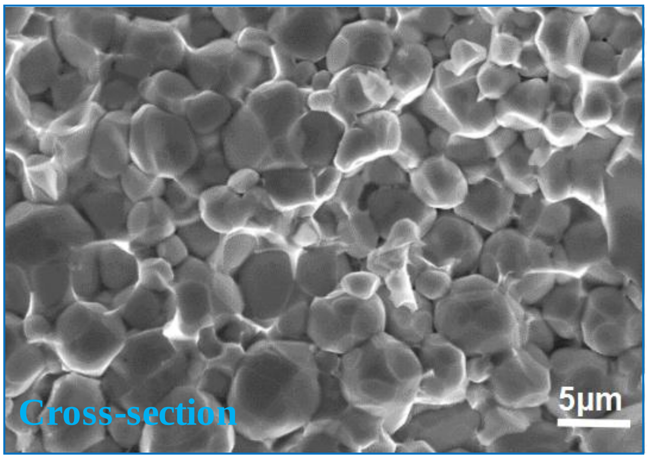

Supplementary Figure 2. Cross-section SEM image of sintered garnet electrolyte, showing no substantial Li_2CO_3 coverage.

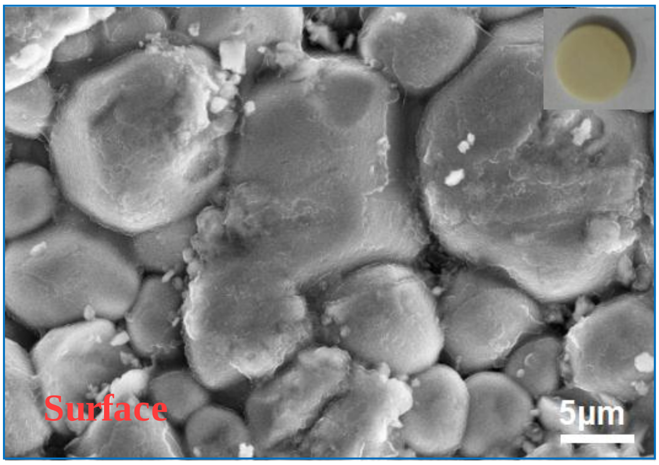

Supplementary Figure 3. Surface morphology of garnet electrolyte, showing a rough texture due to the substantial coverage of Li_2CO_3 layer. Inset: optical image of garnet surface. The surface and profile of sintered ceramic electrolyte show different morphology appearances, and the cross-section of LLZT displays relatively clear grains (Supplementary Figure 2) while LLZT surface is covered by more Li_2CO_3 passivation layers due to longer exposure in air.

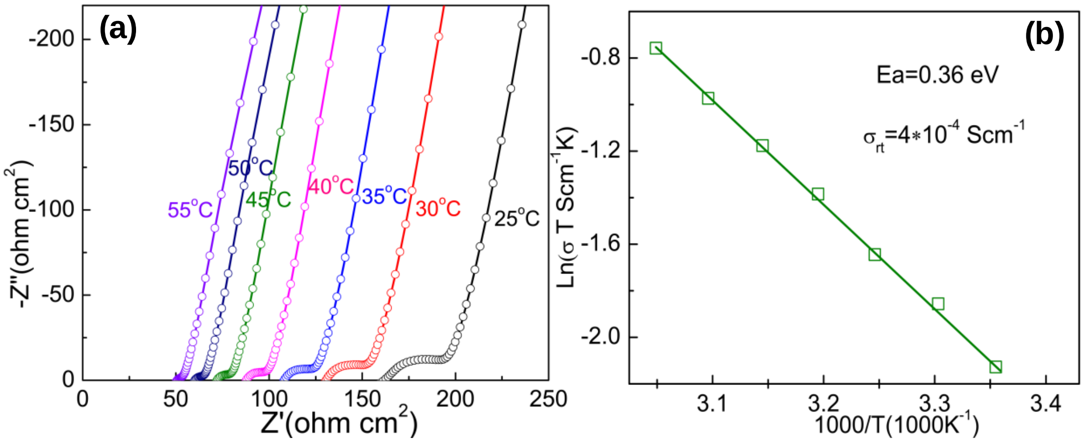

Supplementary Figure 4. (a) Nyquist plots of Ag/LLZT/Ag structure from 25°C to 55°C and (b) Corresponding Arrhenius plots for calculation of activation energy (Ea).

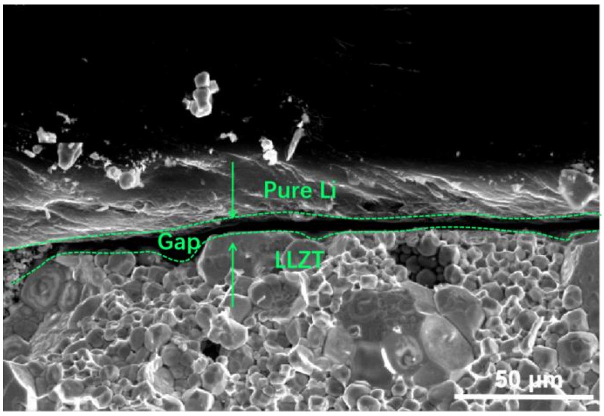

Supplementary Figure 5. Interfacial contact structure of Li anode and unmodified LLZT electrolyte, showing a significant gap.

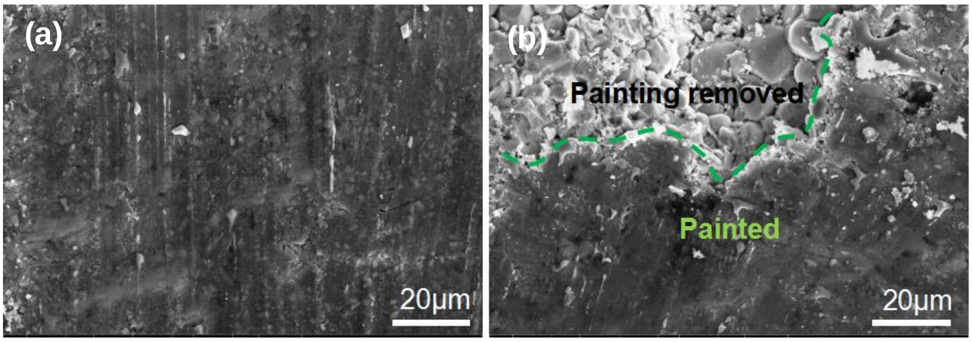

Supplementary Figure 6. SEM images of garnet surface painted by liquid metal (a) with complete coverage and (b) with some region intentionally removed. The painted area is uniform.

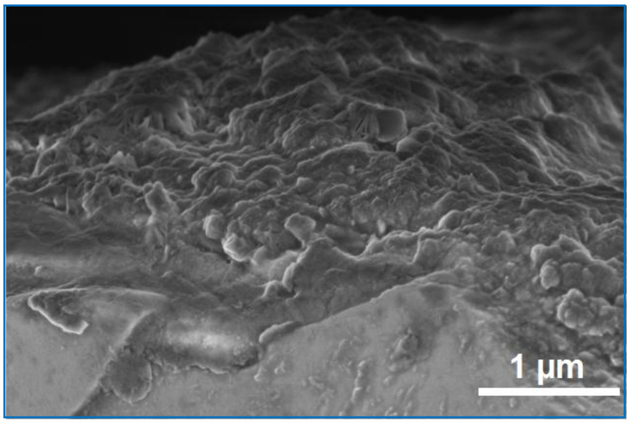

Supplementary Figure 7. Enlarged SEM image focusing on the cross-section of liquid metal painted garnet.

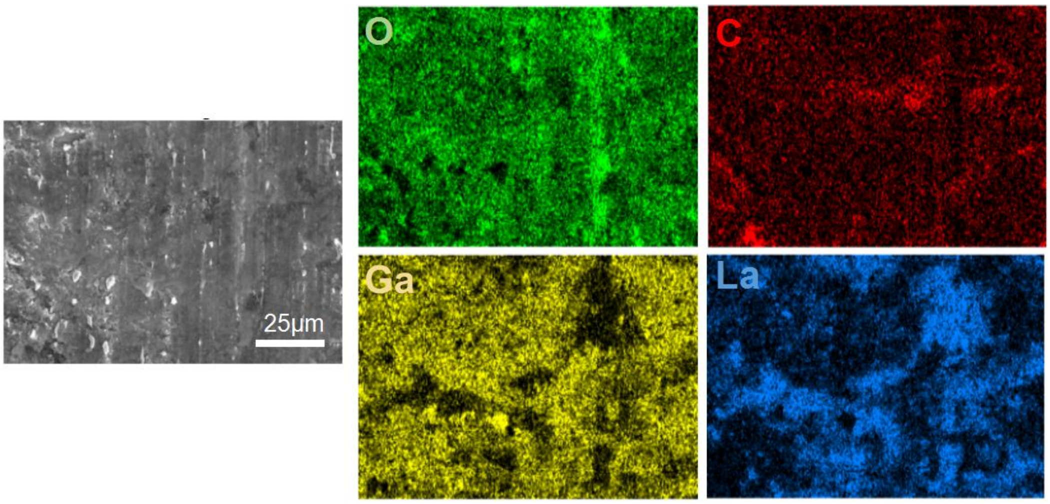

Supplementary Figure 8. EDS mapping result of liquid metal painted garnet surface with the spatial distribution of O, C, Ga, La elements.

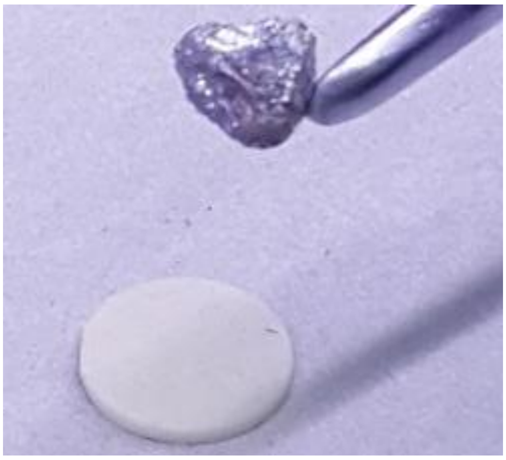

Supplementary Figure 9. Optical image of poor wettability of molten Li on pristine garnet with exposure in air.

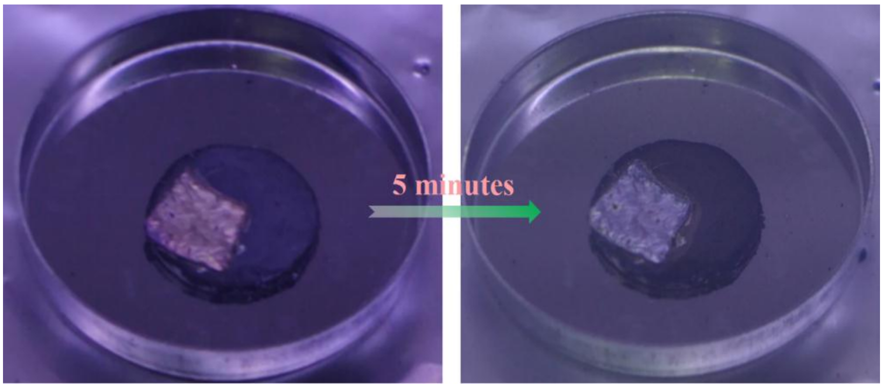

Supplementary Figure 10. Lithiation process of Ga_2O_3 decoration layer at 200°C. The pristine dark (the color of gallium oxide skin) surface turns into yellow (the color of Li) after 5 min.

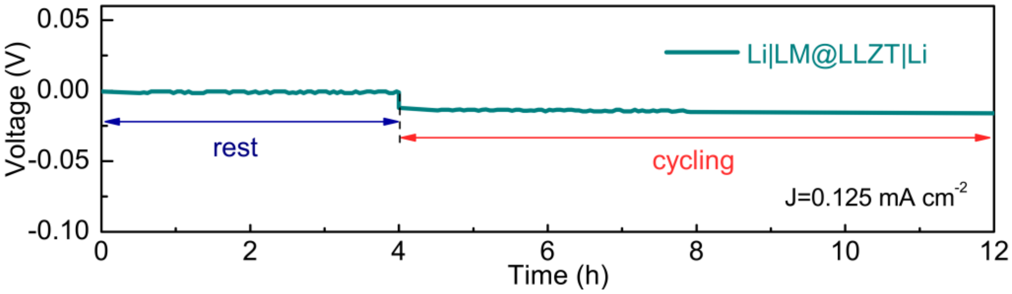

Supplementary Figure 11. Plating curve of Li|LM@LLZT|Li cell at a current density of 0.125 mA cm^{-2} for 8 h.

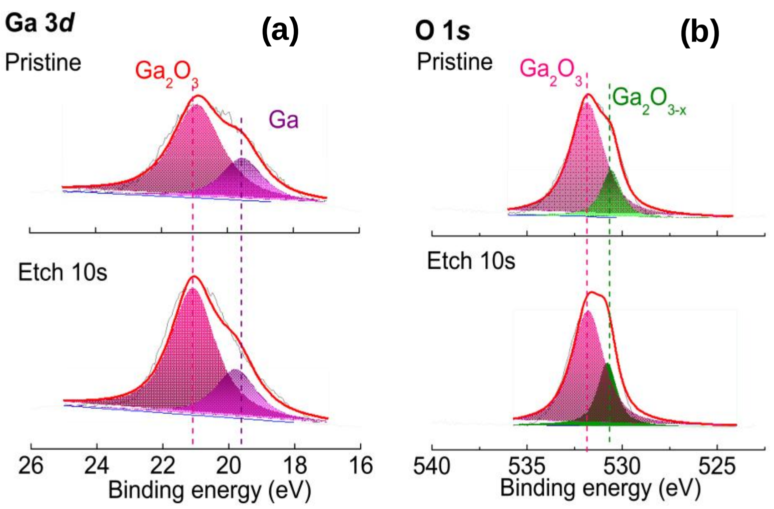

Supplementary Figure 12. XPS spectra of (a) Ga 3*d* and (b) O 1*s* for liquid metal painted garnet (LM@LLZT) surface before etching and after etching for 10s.

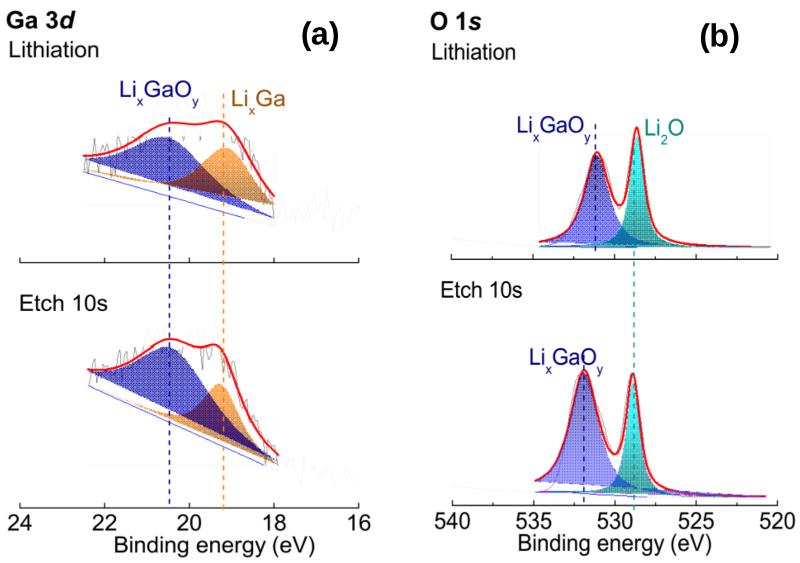

Supplementary Figure 13. XPS spectra of (a) Ga 3*d* and (b) O 1*s* for lithiated LM@LLZT surface before etching and after etching for 10s.

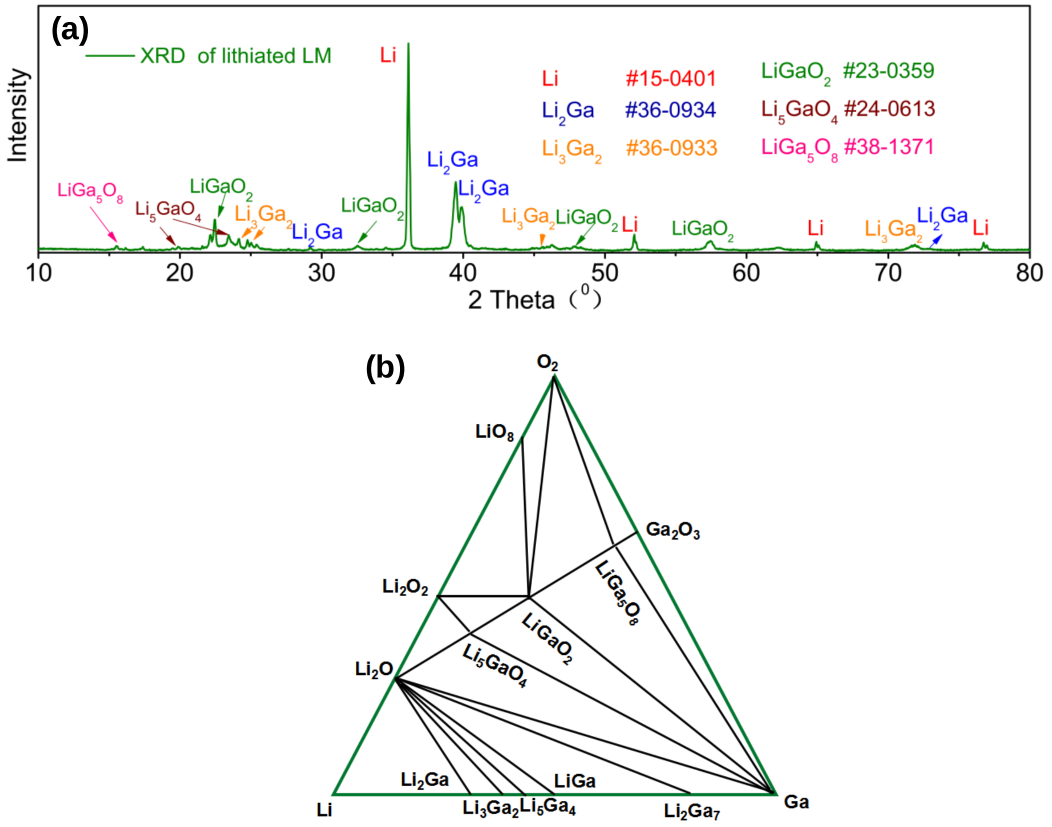

Supplementary Figure 14. (a) Phase distribution of lithiated LM interface layer with oxidized skin obtained from XRD characterization. (b) Phase diagram of Li-Ga-O system. The data is referred from Materials Project database.

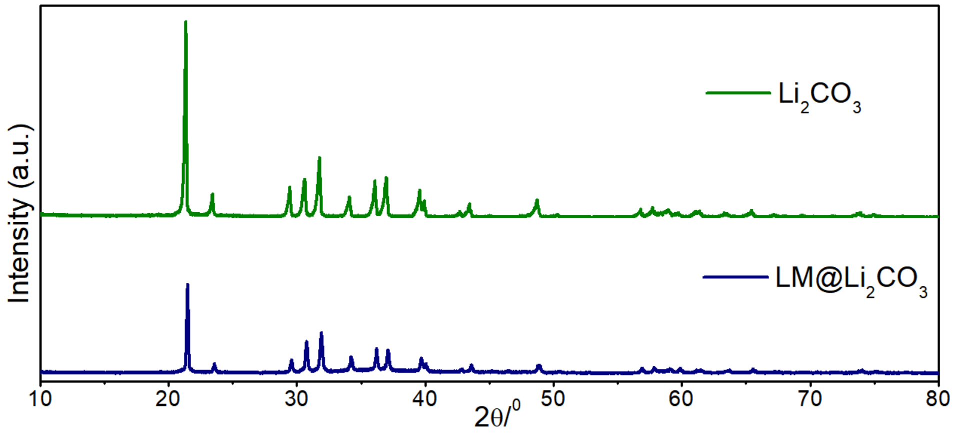

Supplementary Figure 15. Comparison of XRD patterns of liquid metal mixed Li_2CO_3 powder and pure Li_2CO_3 powder.

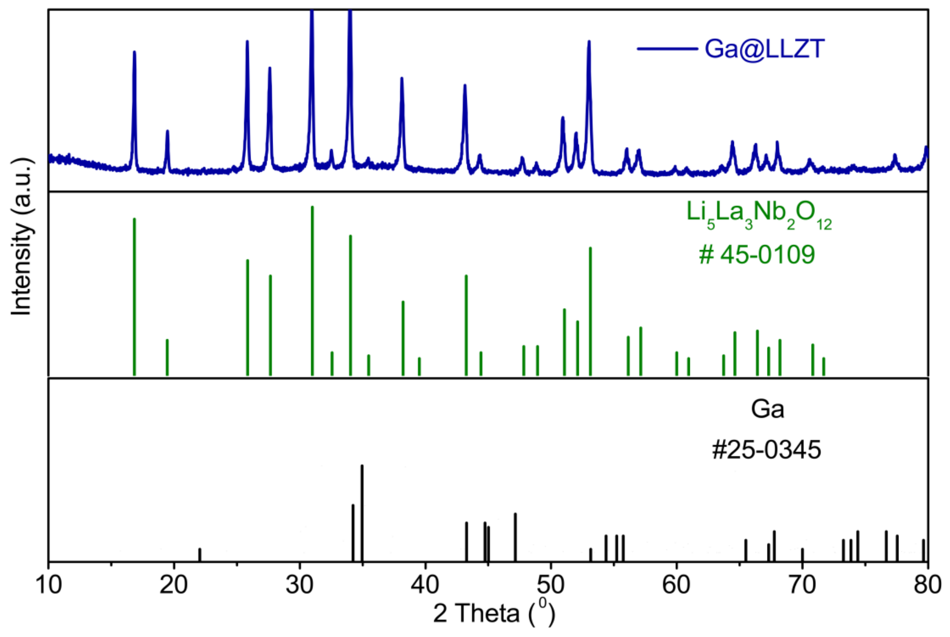

Supplementary Figure 16. XRD pattern of liquid metal mixed LLZT powder.

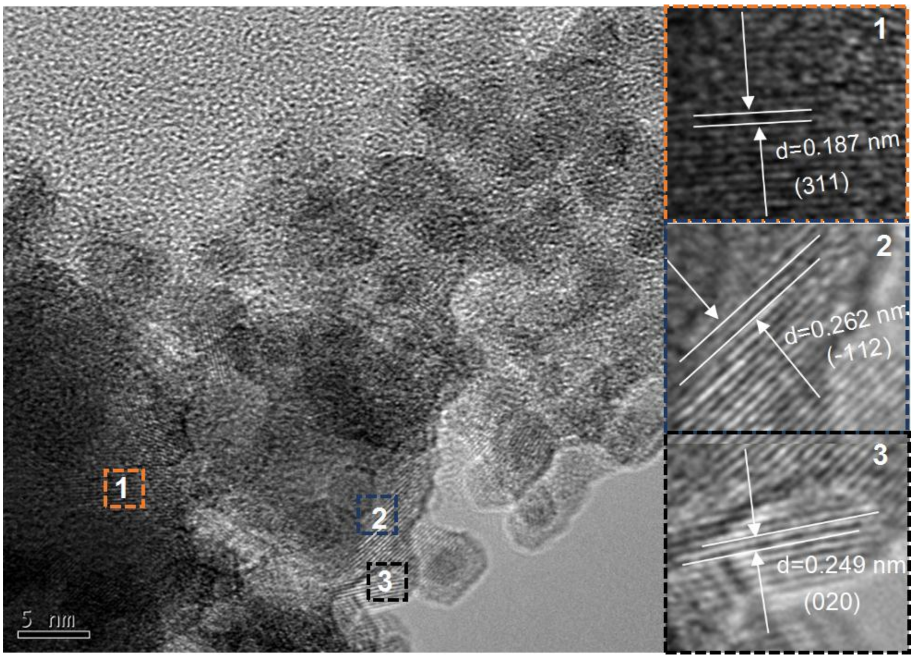

Supplementary Figure 17. HRTEM image of liquid metal mixed Li_2CO_3 powder, showing the Li_2CO_3 nanodomains with typical lattice fringes of (311), (-112), (020) planes with corresponding d-spacings of 0.187, 0.262, 0.249 nm.

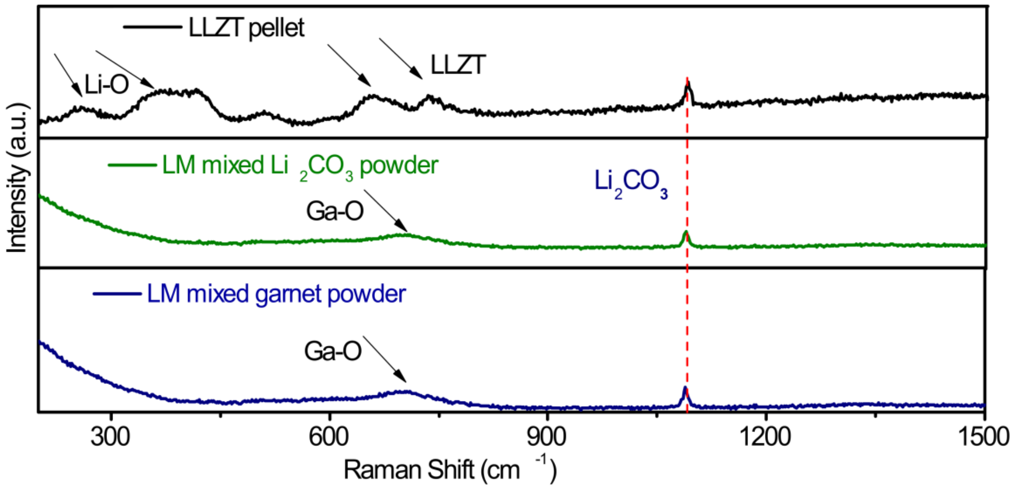

Supplementary Figure 18. Raman spectra of LLZT pellet, liquid metal mixed Li_2CO_3 powder and liquid metal mixed garnet powder.

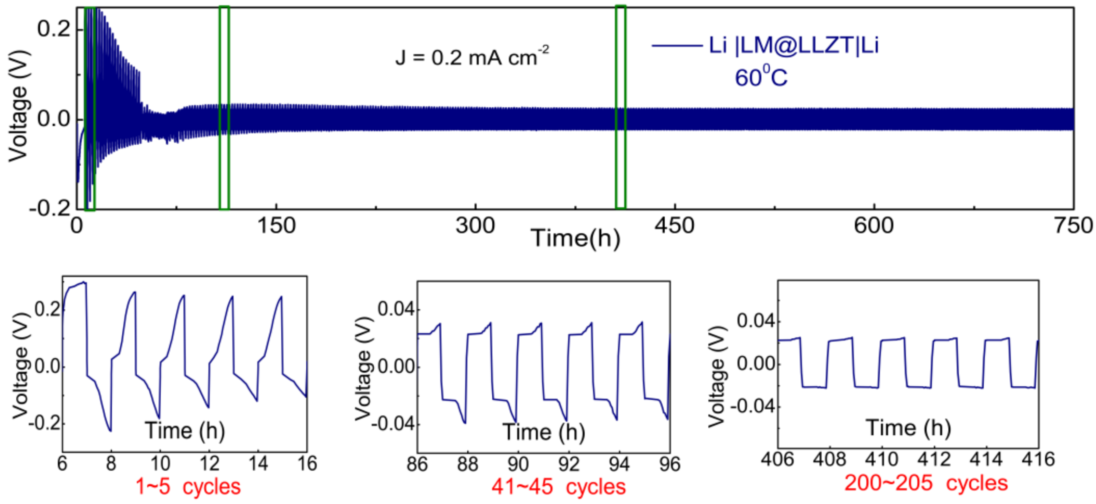

Supplementary Figure 19. Cycling performance of Li|LM@LLZT|Li symmetric cell with pristine Li foil put on liquid metal painted garnet surface (without Li melting pre-treatment) at 0.2 mA cm^{-2}. Below: enlarged voltage profiles from 1 to 5 cycles, from 41 to 45 cycles and from 200 to 205 cycles.

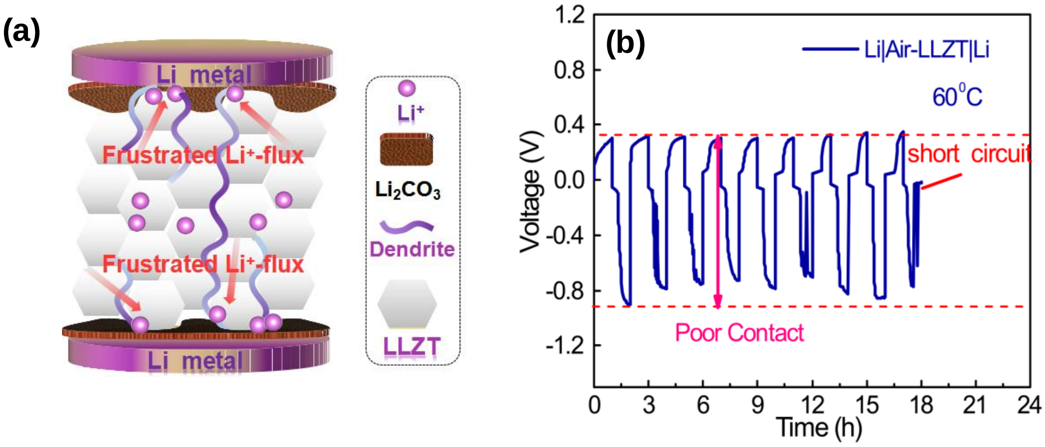

Supplementary Figure 20. (a) Schematic of dendrite production from frustrated Li^+-flux for Li|Air-LLZT|Li structure and (b) voltage curve of symmetric Li|Air-LLZT|Li cell at a current density of 0.1 mA cm^{-2} at 60°C.

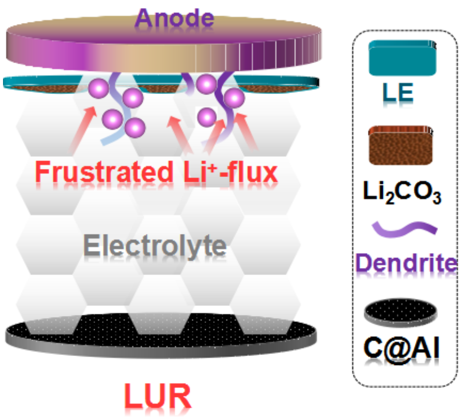

Supplementary Figure 21. Schematic of frustrated Li^+-flux at interface for Li|Air-LLZT|C@Al asymmetric structure and the low utilization ratio (LUR) of Li metal plated on C@Al electrode even under the addition of liquid electrolyte.

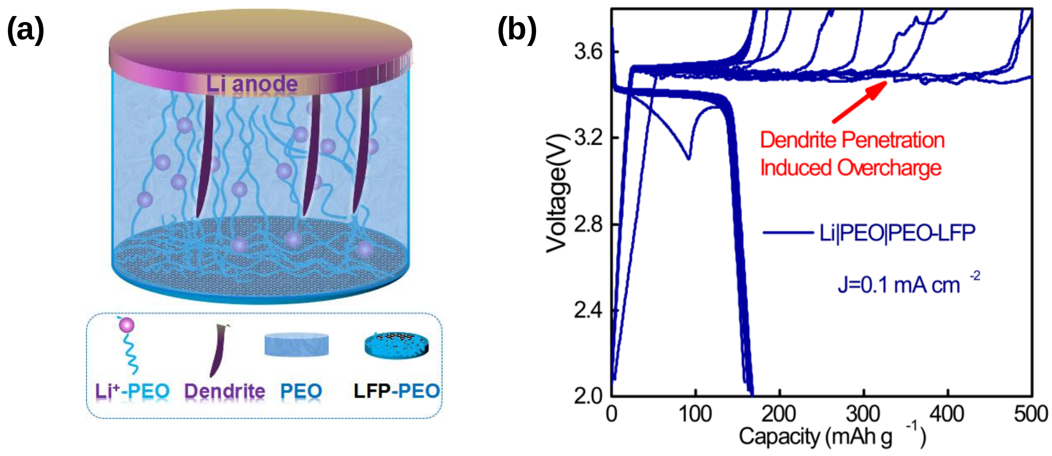

Supplementary Figure 22. (a) Schematic of dendrite growth inside polymer electrolyte for Li|PEO|PEO@LFP structure and (b) voltage-capacity curves of Li|PEO|PEO@LFP full cell at 0.1 mA cm^{-2} and 60°C.

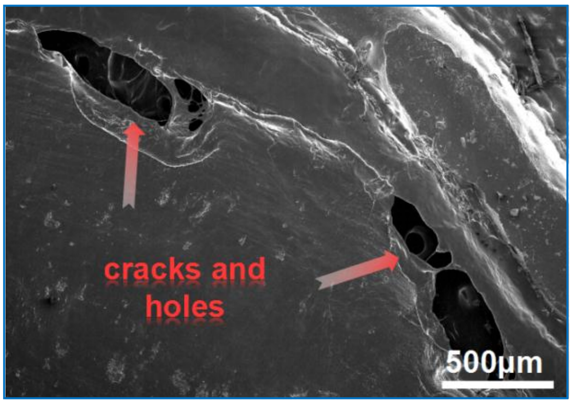

Supplementary Figure 23. SEM image of cycled PEO film from cycled Li|PEO|PEO@LFP cell, showing obvious cracks and holes pierced by dendrite growth.

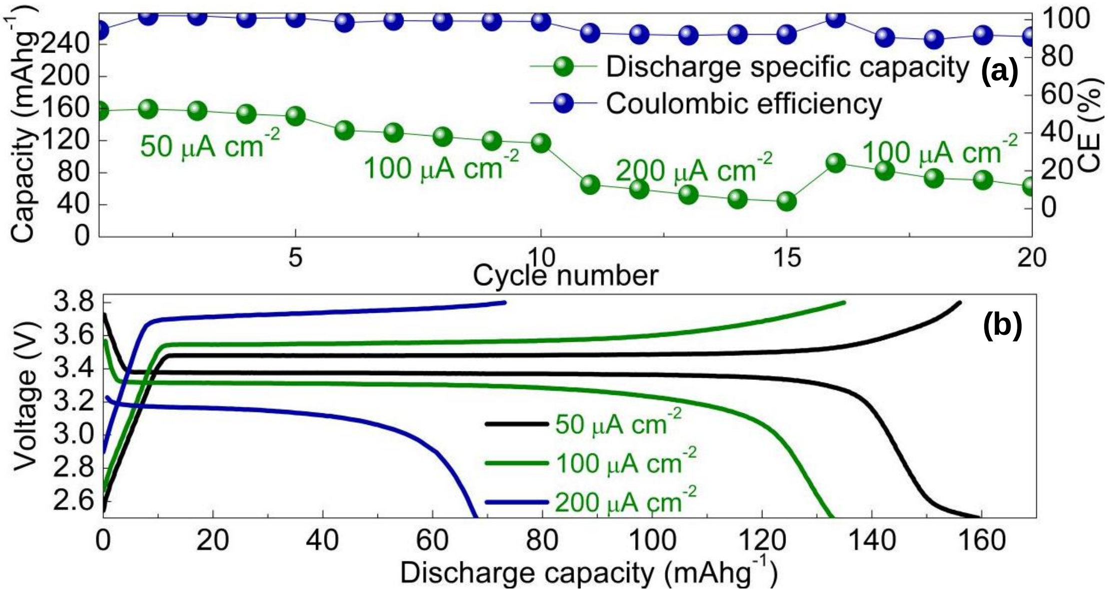

Supplementary Figure 24. (a) Rate performance of Li|LM@LLZT|PEO@LFP solid-state cell operated at room temperature at the current densities from 0.05 to 0.2 mA cm^{-2}. (b) Corresponding voltage profiles of solid-state cell at different current densities. The solid-state cell can be successfully cycled with stable reversible capacities of 157, 120 and 50 mAh g^{-1} at 0.05, 0.1 and 0.2 mA cm^{-2} respectively and the corresponding CEs are close to 100%.

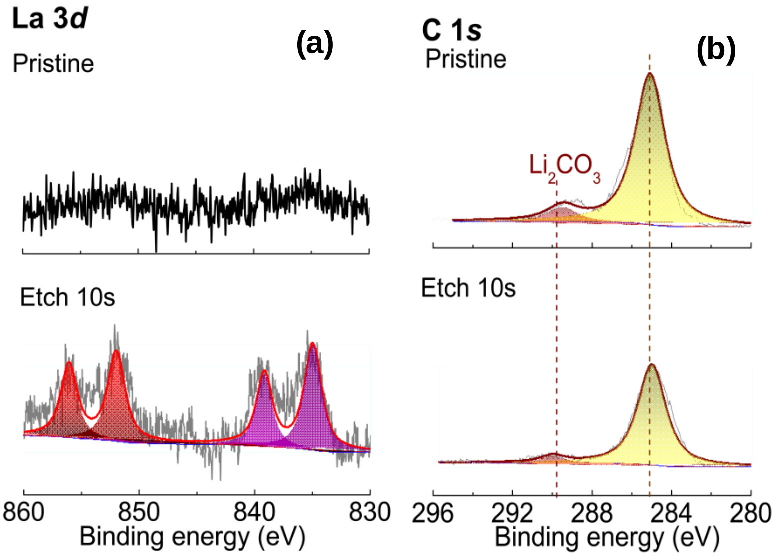

Supplementary Figure 25. XPS spectra of (a) La 3*d* and (b) C 1*s* for liquid metal painted garnet (LM@LLZT) before etching and after etching for 10s.

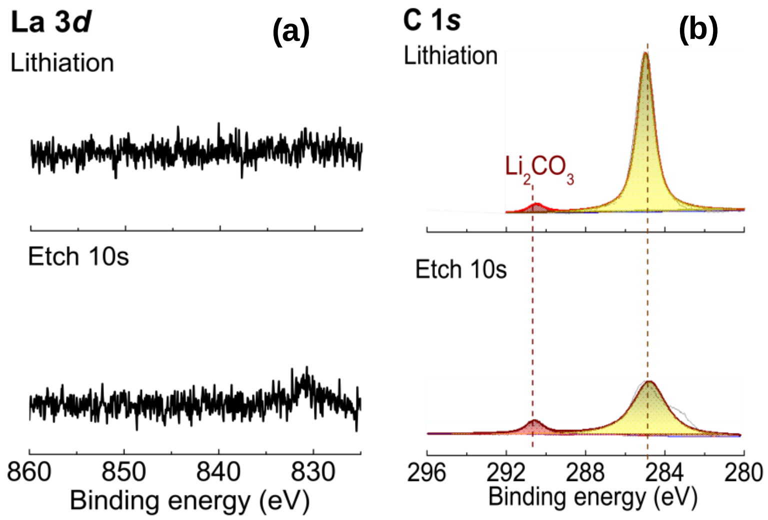

Supplementary Figure 26. XPS spectra of (a) La 3*d* and (b) C 1*s* for lithiated LM@LLZT before etching and after etching for 10s.

Supplementary Table 1. Area specific resistances (R_{int} and R_{surf}), corresponding capacitance values (C), characteristic time constants (τ) and CPE exponent (n) estimated from the impedance spectra of symmetric Li|LLZO|Li or Li|LM@LLZT|Li cells at 25°C and 60°C according to the equivalent circuit in insets of Figure 4a and 4b. (a) Related parameters for interface charge transport process. (b) Related parameters for surface reaction process.

(a) Interface transport

Cell	R_{int} (Ω cm^2)	C [a] (F cm^{-2})	τ [b]	n
Li|LLZT|Li (25°C)	34939	9.1×10^{-9}	0.29 ms	0.79
Li|LLZT|Li (60°C)	543.5	6.8×10^{-9}	3.7 μs	0.77

Li|LM@LLZT|Li (25°C)	39	3.5×10^{-8}	1.37 μs	0.79
Li|LM@LLZT|Li (60°C)	10	1.08×10^{-7}	1.08 μs	0.84

(b) Surface reaction

Cell	R_{surf} (Ω cm^2)	C [a] (F cm^{-2})	τ [b]	n
Li|LLZT|Li (25°C)	3774	2.06×10^{-5}	77.6 ms	0.80
Li|LLZT|Li (60°C)	20	1.6×10^{-6}	32.9 μs	0.84
Li|LM@LLZT|Li (25°C)	5.8	2.8×10^{-7}	1.62 μs	0.85
Li|LM@LLZT|Li (60°C)	4.5	1.7×10^{-7}	0.76 μs	0.87

[a] $C = (R^{1-n}CPE)^{1/n}$

[b] $\tau = RC$

Supplementary Table 2. Comparison of interface resistance and cycling performances of our symmetric Li/Li cells with previous reports based on different modification strategies on garnet electrolyte surface. Pretreatment means the situation of ceramics for use.

Modification Strategy	ASR **(Ω cm^2)**	Pretreatment	Li/Li cell (mAcm^{-2})/h	Ref.
C reduction	28(25°C)	/	0.1/450	1
900°C and Polish	49(25°C)	/	0.9/70	2
Acid(HCl-H_2SO_4)	26(30°C)	Air 30days	0.2/700	3
Au/magnetron sputtering	140(25°C)	180°C/1h	0.03/7	4

Si/PECVD	127	/	0.1/225	5
Mg/magnetron sputtering	70(25°C)	/	0.1/37	6
ZnO/ALD	100	/	0.1/48	7
Sn/magnetron sputtering	46.6	Polish	0.5/500	8
Ag/magnetron sputtering	66	Polish	0.2/100	9
Cu_6Sn_5/magnetron sputtering	236	Polish	0.25/300	10
PEO-PAS	200 Ω (65°C)	/	0.5/10	11
Li-C(graphite)	11	/	0.3/250	12
Li_3N/PECVD	75(25°C)	C Reduction	0.1/210	13
Al/e-beam evaporation	27(60°C)	Polish	0.2/41	14
Al_2O_3/ALD	34(25°C)	Polish	0.2/90	15
Graphite/drawing	13(25°C)	/	0.3/1000	16
MoS_2/coating	7(100°C)	Polish	0.2/40	17
Li-C_3N_4	5.5	/	0.3/300	18
NH_4F	39(25°C)	Air 20days	0.5/200	19
SnN_x/sputtering	82(25°C)	/	0.1/1200	20
LM/Painting	19.5 (RT)<br>5 (60°C)	Air 7days	0.2/9930<br>1/150	This work

Supplementary Table 3. Reaction energies, mutual reaction energies and most

possible phase equilibria for the interfaces between garnet SSE and Li-Ga (or Li-Ga-O) components. Ratio x is the molar fraction of garnet SSE in the pseudo-binary composition. The compositions of garnet SSE and Li-Ga or Li-Ga-O components are already normalized.

(a) Li-Garnet

Ratio of garnet (x)	Reaction energy (meV/atom)	Mutual reaction energy (meV/atom)	Phase equilibria
0.77	-16	-10.61	Li_2O, Zr_4O, La_2O_3
0.78	-16	-10.54	Li_2O, Zr_3O, La_2O_3
1	-7	0	Li_2O, $Li_6Zr_2O_7$, La_2O_3

(b) Li_2Ga-Garnet

Ratio of garnet (x)	Reaction energy (meV/atom)	Mutual reaction energy (meV/atom)	Phase equilibria
0.48	-15	-11.64	Li_2O, Zr_2Ga_3, $LaGa_2$, ZrGa
0.53	-15	-11.29	Li_2O, Zr_3O, La_2O_3
1	-7	0	Li_2O, $Li_6Zr_2O_7$, La_2O_3

(c) Li_3Ga_2-Garnet

Ratio of garnet (x)	Reaction energy (meV/atom)	Mutual reaction energy (meV/atom)	Phase equilibria
0.62	-11	-6.66	Li_2O, $Li_6Zr_2O_7$, $LaGa_2$
1	-7	0	Li_2O, $Li_6Zr_2O_7$, La_2O_3

(d) Li_5Ga_4-Garnet

Ratio of garnet (x)	Reaction energy (meV/atom)	Mutual reaction energy (meV/atom)	Phase equilibria
0.25	-6	-4.25	Li_2O, $Li_6Zr_2O_7$, $LaGa_2$
1	-7	0	Li_2O, $Li_6Zr_2O_7$, La_2O_3

(e) $LiGaO_2$-Garnet

Ratio of garnet (x)	Reaction energy (meV/atom)	Mutual reaction energy (meV/atom)	Phase equilibria

0.96	-9	-2.28	Li_5GaO_4, $Li_6Zr_2O_7$, La_2O_3
1	-7	0	Li_2O, $Li_6Zr_2O_7$, La_2O_3

(f) $LiGa_5O_8$-Garnet

Ratio of garnet (x)	Reaction energy (meV/atom)	Mutual reaction energy (meV/atom)	Phase equilibria
0.316	-84	-81.79	$LiGaO_2$, $La_2Zr_2O_7$, $La_3Ga_5O_{12}$
0.348	-90	-87.56	$LiGaO_2$, $La_2Zr_2O_7$, $La_4Ga_2O_9$
0.364	-89	-86.45	$LiGaO_2$, $La_2Zr_2O_7$, La_2O_3
0.8	-32	-29.79	$LiGaO_2$, $Li_6Zr_2O_7$,La_2O_3
0.96	-14	-7.28	Li_5GaO_4, $Li_6Zr_2O_7$,La_2O_3
1	-7	0	Li_2O, $Li_6Zr_2O_7$, La_2O_3

Supplementary References

1. Li, Y. et al. Garnet electrolyte with an ultralow interfacial resistance for Li-metal batteries. *J. Am. Chem. Soc.* **140**, 6448-6455 (2018).

2. Wu, J. F. et al. In-situ formed shields enabling Li_2CO_3-free solid electrolytes: A new route to uncover the intrinsic lithiophilicity of garnet electrolytes for dendrite-free Li-metal batteries. *ACS Appl. Mater. Interfaces* **11**, 898-905 (2018).

3. Huo, H. et al. In-situ formed Li_2CO_3-free garnet/Li interface by rapid acid treatment for dendrite-free solid-state batteries. *Nano Energy* **61**, 119-125 (2019).

4. Alexander, G. V., Patra, S., Raj, S. V. S., Sugumar, M. K., Din, M. M. U. & Murugan, R. Electrodes-electrolyte interfacial engineering for realizing room temperature lithium metal battery based on garnet structured solid fast Li^+ conductors. *J. Power Sources* **396**, 764-773 (2018).

5. Luo, W. et al.Transition from superlithiophobicity to superlithiophilicity of garnet solid-state electrolyte. *J. Am. Chem. Soc.* **138**, 12258-12262 (2016).

6. Fu, K. et al. Transient behavior of the metal interface in lithium metal-garnet batteries. *Angew. Chem. Int. Ed.* **56**, 14942-14947 (2017).

7. Wang, C. et al. Conformal, nanoscale ZnO surface modification of garnet-based solid-state electrolyte for lithium metal anodes. *Nano. Lett.* **17**, 565-571 (2017).

8. He, M., Cui, Z., Chen, C., Li, Y. & Guo, X. Formation of self-limited, stable and conductive interfaces between garnet electrolytes and lithium anodes for reversible lithium cycling in solid-state batteries. *J. Mater. Chem. A* **6**, 11463-11470 (2018).

9. Feng, W., Dong, X., Li, P., Wang, Y. & Xia, Y. Interfacial modification of Li/garnet electrolyte by a lithiophilic and breathing interlayer. *J. Power Sources* **419**, 91-98 (2019).

10. Feng, W. et al. Building an interfacial framework: Li/garnet interface stabilization through a Cu_6Sn_5 Layer. *ACS Energy Lett.* **4**, 1725-1731 (2019).

11. Zhou, W. et al. Polymer lithium-garnet interphase for an all-solid-state rechargeable battery. *Nano Energy* **53**, 926-931 (2018).

12. Duan, J. et al. Lithium-graphite paste: an interface compatible anode for solid-state batteries. *Adv. Mater.* **31**, 1807243 (2019).

13. Xu, H. et al. Li_3N-Modified garnet electrolyte for all-solid-state lithium metal batteries operated at 40 ℃. *Nano. Lett.* **18**, 7414-7418 (2018).

14. Fu, K. K. et al. Toward garnet electrolyte-based Li metal batteries: An ultrathin, highly effective, artificial solid-state electrolyte/metallic Li interface. *Sci. Adv.* **3**, e1601659 (2017).

15. Han, X. G. et al. Negating interfacial impedance in garnet-based solid-state Li metal batteries. *Nat. Mater.* **16**, 572-579 (2017).

16. Shao, Y. et al. Drawing a soft interface: an effective interfacial modification strategy for garnet-type solid-state Li batteries. *ACS Energy Lett.* **3**, 1212-1218 (2018).

17. Fu, J. et al. In situ formation of a bifunctional interlayer enabled by a conversion reaction to initiatively prevent lithium dendrites in a garnet solid electrolyte. *Energy Environ. Sci.* **12**, 1404-1412 (2019).

18. Huang, Y. et al. Graphitic carbon nitride ($g\text{-}C_3N_4$): An interface enabler for

solid-state lithium metal batteries. *Angew. Chem. Int. Ed.* **59**, 3699-3704 (2020).

19. Duan, H. et al. Building an air stable and lithium deposition regulable garnet interface from moderate-temperature conversion chemistry. *Angew. Chem. Int. Ed.* **59**, 2-9 (2020).

20. Shi, K. et al. In-situ construction of an ultra-stable conductive composite interface for high-voltage all-solid-state lithium metal batteries. *Angew. Chem. Int. Ed.* DOI: 10.1002/anie.202000547 (2020).
